# Proton pump inhibitors and gut microbiota dysbiosis: insights into the pathogenesis of ulcerative colitis

**DOI:** 10.3389/fmicb.2025.1657865

**Published:** 2025-10-30

**Authors:** Feng Cao, Zhenxin Liu, Shuo Liu, Yanwei Liu, Yong Qi, Yunsheng Cheng, Yong Wang, Yanyan Xu

**Affiliations:** ^1^Department of General Surgery, Second Affiliated Hospital of Anhui Medical University, Hefei, Anhui, China; ^2^Department of General Surgery, University Hospital RWTH Aachen, Aachen, Germany; ^3^Ningxia Medical University College of Traditional Chinese Medicine, Ningxia, China

**Keywords:** ulcerative colitis, proton pump inhibitors, omeprazole, gut inflammation, gut microbiota dysbiosis

## Abstract

**Background:**

Ulcerative colitis (UC) is a chronic, relapsing inflammatory bowel disease characterized by continuous mucosal inflammation of the colon and rectum. The global prevalence of UC has been rising steadily, and accumulating evidence suggests a potential association between proton pump inhibitor (PPIs) use and UC development. Nevertheless, the precise role of PPIs in the pathogenesis and clinical course of UC remains unclear.

**Methods:**

The C57BL/6J mice were administered saline, omeprazole (OME) and dextran sulfate sodium to establish control, PPIs-treated and UC models, respectively. The fecal samples were subjected to high-throughput sequencing of the V3-V4 hypervariable regions of the 16S rRNA gene. Taxonomic annotation was performed using Mothur software to evaluate microbial diversity and abundance. Principal coordinate analysis, linear discriminant analysis effect size, and functional enrichment analyses were also conducted.

**Results:**

Alpha and beta diversity analyses showed that the richness and diversity of the gut microbiota in the PPI and UC groups were significantly lower than those in the control group (*p* < 0.05). At the family and genus levels, the UC group was dominated by *Bacteroides*, while the PPIs group exhibited enrichment of *Eisenbergiella* and *Prevotella*. Furthermore, functional enrichment analysis demonstrated that the gut microbiota in the PPI group was predominantly enriched in functions related to cell wall and membrane structure biogenesis, whereas the UC group was enriched in energy metabolism.

**Conclusion:**

Long-term PPI exposure profoundly alters the gut microbiota, characterized by reduced microbial diversity and enrichment of pro-inflammatory taxa. These findings highlight the contribution of PPIs to gut microbiota dysbiosis and UC pathogenesis, emphasizing the need for further research on microbiota–immunity interactions and for the development of targeted strategies to mitigate PPI-related adverse effects.

## 1 Introduction

Ulcerative colitis (UC) is a chronic, relapsing inflammatory bowel disease (IBD) characterized by continuous mucosal inflammation of the colon and rectum (Voelker, 2024). Over the past decades, the global prevalence and incidence of UC have increased markedly, particularly in newly industrialized regions (Dharni et al., 2024). The persistent symptoms of UC, such as bloody diarrhea, abdominal pain, and weight loss, impose a substantial clinical and societal burden and are associated with an elevated risk of long-term complications, including colorectal cancer and extraintestinal manifestations. Despite advances in our understanding, the etiology of UC and the mechanisms underlying its chronic and heterogeneous nature remain incompletely elucidated (Kobayashi et al., 2020). Proton pump inhibitors (PPIs), among the most widely prescribed medications worldwide, have attracted increasing attention for their potential impact on intestinal homeostasis. Emerging evidence indicates that PPI use may alter the gut microbiota and modulate mucosal immunity, thereby contributing to the development and progression of UC (Singh et al., 2023; Shah et al., 2017).

PPIs effectively inhibit gastric acid secretion and are commonly used to treat peptic ulcers, gastroesophageal reflux, Zollinger-Ellison syndrome, upper gastrointestinal hemorrhage, and *H. pylori* infection (Abrahami et al., 2022; Clarke et al., 2022). However, many studies have shown that long-term use of PPIs may lead to a variety of adverse effects such as fractures, *Clostridium difficile* infection, colorectal cancer, and stroke (Abrahami et al., 2022; Yang et al., 2021; Freedberg et al., 2017). Recent studies have shown that PPIs use is significantly associated with an increased risk of IBD. Xia et al. (2021) found that regular or frequent use of PPIs significantly increased the risk of UC by pooled analysis of three prospective study cohorts. Similarly, in patients with UC, PPI exposure can induce disease exacerbation and increase the incidence of IBD-related adverse events, such as hospitalization or surgery (Shah et al., 2017). In addition, the use of PPIs is associated with decreased remission and increased hospitalization rates in IBD patients treated with infliximab (Lu et al., 2021). However, the potential mechanisms through which PPIs influence IBD remain largely unexplored.

Recent studies have shown that PPI-mediated gastric pH elevation can increase the migration of bacteria from the oral cavity to the intestinal lumen (Macke et al., 2020). This process reduces gut microbiota diversity and increases the abundance of potential oral pathogens (Hopkins et al., 2022). In addition, PPIs may increase colonic mucosal permeability (Takashima et al., 2020). Coincidentally, gut microbiota dysbiosis and gut barrier disruption promote bacterial infection in the intestine, a central factor in the pathogenesis of UC (Azimi et al., 2018). And these alterations are also key risk factors for adverse outcomes in patients receiving PPI therapy (Naito et al., 2018). Furthermore, the gut microbiota of PPIs users and IBD patients share certain characteristics, manifested by a decrease in the diversity and abundance of the anti-inflammatory microbiota Faecalibacterium (Carr, 2018). These findings suggest that the PPI-mediated pathogenesis of UC may be closely related to gut microbiota dysbiosis.

In summary, although PPIs have been implicated in the onset and progression of UC, current evidence remains limited. In this study, we conducted an exploratory animal experiment to investigate the effects of PPI exposure on gut inflammation and gut microbiota dysbiosis in models of UC, with particular attention to the potential disruption of microbial diversity and the enrichment of pro-inflammatory taxa that may exacerbate mucosal inflammation and disease progression. These findings provide an experimental basis for elucidating the role of PPIs in UC and may inform future clinical applications and therapeutic strategies.

## 2 Materials and methods

### 2.1 Establishment of the PPI and UC mouse models

#### 2.1.1 Ethical approval

All mice were purchased from Hangzhou Ziyuan Laboratory Animal Technology Co., Ltd., and all experimental procedures were approved by the Experimental Animal Ethics Committee of Anhui Medical University (protocol no. LLSC20230782).

#### 2.1.2 Dose optimization of omeprazole

Male C57BL/6 J mice (6–8 weeks old) were acclimatized for 1 week under standard laboratory conditions (room temperature, 12-h light/dark cycle). To determine the optimal dosage of omeprazole (OME; MedChemExpress, CAS: 73590–58-6), mice (*n* = 6 per group) received oral gavage of OME at doses of 0, 1, 5, 10, 15, or 20 mg/kg/day for 8 weeks. Based on induction results, 10 mg/kg/day was selected for subsequent modeling.

#### 2.1.3 Animal model and grouping

Mice were randomly assigned to three groups: control (*n* = 5), PPI-treated (*n* = 5), and ulcerative colitis (UC; *n* = 10). The control group (saline-treated) was used to establish baseline microbial and inflammatory readouts. The PPI-treated group received omeprazole (OME) at 10 mg/kg/day by oral gavage for 8 weeks, allowing evaluation of the effects of chronic PPI exposure in the absence of DSS. The UC group, induced with dextran sulfate sodium (DSS; MP Biomedicals, CAS: 9011-18-1), served as the positive control to validate inflammation-related outcomes. UC was induced by administering 2.5% DSS in drinking water for 7 consecutive days, followed by 7 days of regular water. This two-cycle regimen was repeated, yielding a total induction period of 28 days.

### 2.2 Body weight monitoring and sample collection

Throughout the experimental period, mouse body weights were recorded daily prior to administration to monitor weight changes. At the end of the induction phase (8 weeks for the PPI group and 28 days for the UC group), mice were anesthetized, and blood was collected from the abdominal aorta. Serum was isolated by centrifugation and stored at −20 °C for subsequent inflammatory cytokine analysis. The colonic tissue was harvested and washed with normal saline. Each sample was fixed in 10% neutral formalin solution for histological examination and immunohistochemistry. Fresh fecal samples were collected, flash-frozen in liquid nitrogen, and stored at −80 °C until further processing for microbiota analysis.

### 2.3 Enzyme-linked immunosorbent assay (ELISA)

Serum concentrations of interleukin (IL)-1β, IL-6, tumor necrosis factor-alpha (TNF-*α*), IL-10, and myeloperoxidase (MPO) were determined using enzyme-linked immunosorbent assay kits (ELISA, BosterBio, Wuhan, China), kits (BosterBio, Wuhan, China), following the manufacturer’s protocols. Briefly, antibody-coated 96-well plates were incubated with serum samples at 37 °C for 90 min. After washing, biotin-labeled detection antibodies were added and incubated for 60 min at 37 °C. Optical density (OD) was measured at 560 nm using a microplate reader (Molecular Devices), and cytokine concentrations were calculated based on standard curves.

### 2.4 Hematoxylin–eosin (HE) staining and immunohistochemistry (IHC)

Colon tissues previously fixed in formaldehyde solution were paraffin-embedded, sectioned, stained with HE, and examined microscopically. Histological scoring was performed to evaluate crypt architecture destruction and inflammatory cell infiltration according to the following criteria:

Crypt architecture destruction score:

- Score 0: normal crypt arrangement with uniform density;
- Score 1: mild crypt distortion with partial widening of crypt spacing (<30% of crypts affected);
- Score 2: moderate crypt atrophy or branching, 30–60% of crypts abnormal, with goblet cell depletion;
- Score 3: severe crypt destruction (>60%), irregular hyperplasia or pseudopolyps present, with near-complete loss of goblet cells.

Inflammatory cell infiltration score:

- Score 0: none or few lymphocytes in the lamina propria, with no neutrophils;
- Score 1: increased lymphocytes in the lamina propria with occasional neutrophils (<5 per high-power field, HPF);
- Score 2: neutrophil infiltration extending to the muscularis mucosa, with pericryptal aggregation (5–15 neutrophils/HPF);
- Score 3: diffuse infiltration of numerous neutrophils throughout the mucosa, with crypt abscess formation (>15 neutrophils/HPF).

In addition, IHC was performed on colon sections to assess interleukin-6 (IL-6) expression. Images were analyzed using Image-Pro Plus 6.0 software, and the mean optical density (AOD) from three randomly selected fields was calculated. Higher AOD values were considered indicative of more severe tissue inflammation.

### 2.5 Stool DNA extraction and quality assessment

Genomic DNA was extracted from fecal samples using the QIAamp® DNA Stool Mini Kit (Qiagen, Hilden, Germany) according to the manufacturer’s instructions. DNA integrity was assessed by agarose gel electrophoresis, and concentration and purity were evaluated using a NanoDrop 2000 spectrophotometer (10 × Genomics, USA).

### 2.6 16S rRNA gene amplification and high-throughput sequencing

The V3–V4 hypervariable regions of the bacterial 16S rRNA gene were amplified in triplicate using high-fidelity polymerase chain reaction (high-fidelity PCR) with the primers 341F (5′-CCTACGGGNGGCWGCAG-3′) and 806R (5′-GACTACHVG GGTATCTAATCC-3′). The specificity of amplification was confirmed by agarose gel electrophoresis. Amplicons were purified using the Agencourt AMPure XP Kit (Beckman Coulter, USA), and sample-specific index sequences were added during a second round of PCR. Library quality was assessed using a Qubit 3.0 Fluorometer (Invitrogen, Thermo Fisher Scientific, USA) and an Agilent 2100 Bioanalyzer (Agilent Technologies, USA). The pooled libraries were sequenced on an Illumina NovaSeq 6000 platform (Illumina, USA) to generate 2 × 250 bp paired-end reads.

### 2.7 Bioinformatics analysis

Raw sequencing reads were subjected to quality control and filtering as follows: (1) low-quality reads (average quality score <20), reads with adapters, or reads <100 bp were removed using TrimGalore; (2) paired-end reads were merged using FLASH (v1.2.11); (3) sequences with ambiguous bases or homopolymers >6 bp were filtered out using Mothur; (4) low-complexity reads were excluded to obtain high-quality clean reads. Chimeric sequences were identified using the gold.fa reference database^1^ and removed. Clean reads were clustered into operational taxonomic units (OTUs) at 97% similarity using UPARSE, and taxonomic annotation was performed with the Ribosomal Database Project (RDP) Release 9 (201203) via Mothur.

### 2.8 Diversity and statistical analysis

Alpha diversity indices (e.g., Shannon, Simpson) and rarefaction curves were calculated using Mothur. Beta diversity was assessed using Bray–Curtis hierarchical clustering, unweighted pair-group method with arithmetic mean (UPGMA), and Jaccard-based principal coordinate analysis (PCoA) via the Vegan package (v3.3.1) in R. Redundancy analysis (RDA) was conducted using Canoco for Windows 4.5 (Microcomputer Power, NY, USA), with significance assessed by Monte Carlo permutation tests (*n* = 499).

All statistical analyses were performed using SPSS software (version 22.0; IBM Corp., Armonk, NY, USA). Based on the normality test of the original data, one-way analysis of variance (ANOVA) or the Kruskal–Wallis test was used to compare groups. Correlations were analyzed using Spearman’s rank correlation coefficient. *p*-values were adjusted for multiple testing using the Bonferroni false discovery rate (FDR) method, and FDR-adjusted *p*-values <0.05 were considered statistically significant.

## 3 Results

### 3.1 Mouse PPI model induction

**Figure 1:**
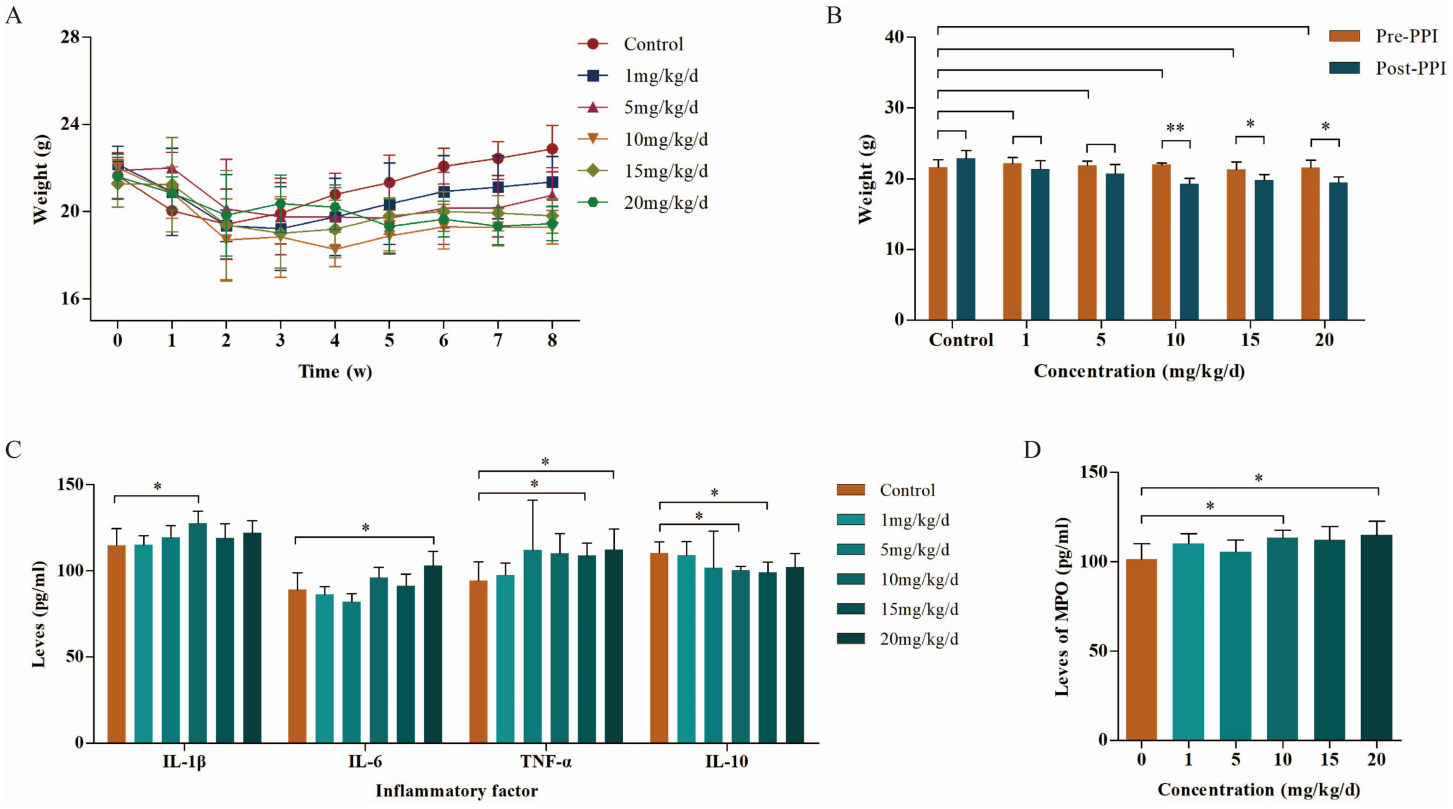
Body weight and inflammatory factors in mice during the PPI-induced period (*n* = 6 per group). **(A)** Weight change curves. **(B)** Body weight of mice induced by different concentrations of PPIs. **(C)** Levels of inflammatory factors in abdominal aorta of mice induced by different concentrations of PPIs. **(D)** Levels of MPO in abdominal aorta of mice induced by different concentrations of PPIs. *Indicates 0.01 ≤ *p* < 0.05, **indicates *p* < 0.01.

Body weight changes in mice in each group after OME administration at different concentrations are shown in Figures 1A,B. Weight in all groups decreased during the first 2 weeks. Starting from the 3rd week, the weight of the control group gradually recovered, while that of the PPI group recovered somewhat, and the overall trend was downward. When the OME concentration was 10 mg/kg/d, the weight loss was the highest (22.00 ± 0.20 g vs. 19.28 ± 0.77 g, *p* < 0.001), and above 10 mg/kg/d, the weight loss did not change with an increase in OME concentration (15 mg/kg/d: 21.28 ± 1.08 g vs. 19.80 ± 0.79 g, *p* = 0.022; 20 mg/kg/d: 21.60 ± 1.03 g vs. 19.45 ± 0.79 g, *p* = 0.002).

The results of ELISA showed that the levels of pro-inflammatory factors IL-1β, IL-6, and TNF-*α* in the blood were increased in a concentration-dependent manner with OME, while levels of the anti-inflammatory factor IL-10 were opposite. This correlation peaked at an OME concentration of 10 mg/kg/day (Figure 1C). MPO, another factor that determines the degree of the inflammatory reaction, showed similar changes to pro-inflammatory factors (10 mg/kg/d: 101.27 ± 8.75 pg./mL vs. 113.59 ± 4.17 pg./mL, *p* = 0.022; 15 mg/kg/d: 101.27 ± 8.75 pg./mL vs. 112.17 ± 7.58 pg./mL, *p* = 0.068; 20 mg/kg/d: 101.27 ± 8.75 pg./mL vs. 114.88 ± 7.78 pg./mL, *p* = 0.032; Figure 1D). Combined with body weight and inflammatory factor levels, we selected 10 mg/kg/day as the optimal induction concentration, which was adopted in subsequent studies.

### 3.2 OME and DSS induced colonic inflammation

**Figure 2:**
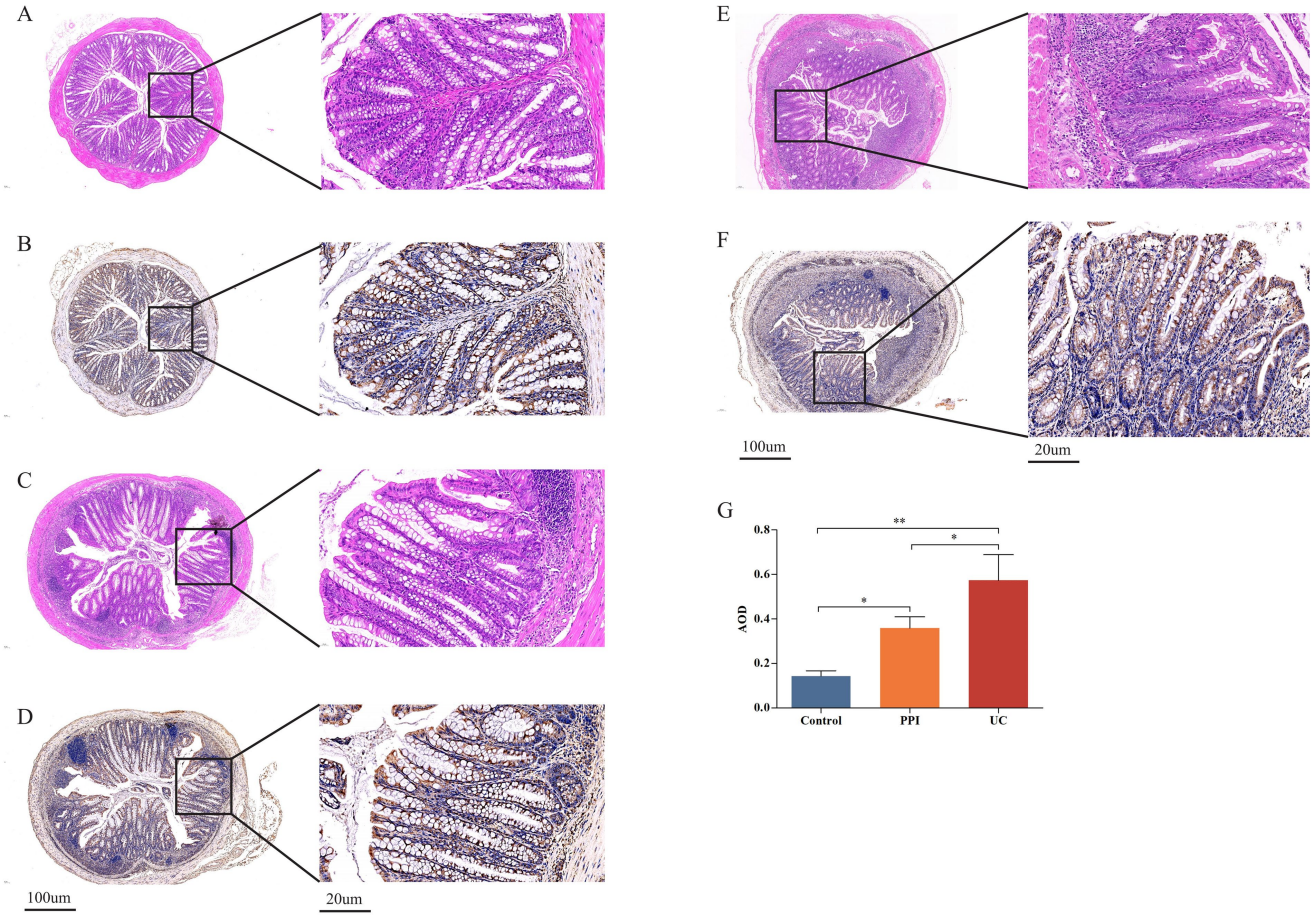
Colonic tissue pathological examination among control (*n* = 5), PPI (*n* = 5) and UC (*n* = 10) groups. **(A)** Representative images of crypt destruction and inflammatory infiltration in control group. **(B)** Representative images of crypt destruction and inflammatory infiltration in PPI group. **(C)** Representative images of crypt destruction and inflammatory infiltration in UC group. **(D)** Representative images of IL-6 expression in control group. **(E)** Representative images of IL-6 expression in PPI group. **(F)** Representative images of IL-6 expression in UC group. **(G)** The relative statistical analysis of IHC. *Indicates 0.01 ≤ *p* < 0.05, **indicates *p* < 0.01.

Colonic inflammation was evaluated by HE staining and IHC. HE staining showed that crypt destruction and inflammatory infiltration scores were both 0 in the control group, 2 in the PPI group, and 3 in the UC group (Figures 2A–C). Immunohistochemical analysis demonstrated a stepwise increase in the AOD values among the control, PPI, and UC groups, with statistically significant differences (0.14 ± 0.02 vs. 0.36 ± 0.05 vs. 0.57 ± 0.12, *p* = 0.001; Figures 2D–G). These findings suggest that long-term OME administration may elicit colonic inflammatory responses comparable to those observed in DSS-induced colitis.

### 3.3 OME and DSS induce gut microbiota dysbiosis

#### 3.3.1 Sequencing depth and species diversity

The rarefaction curve tends to flatten with an increase in extracted sequences, indicating that the sample sequencing is reasonable and the sequencing depth is basically covered, and it can be used for subsequent analysis (Supplementary Figure S1A). In addition, the species accumulation curves gradually tends to flatten, indicating that all samples are sufficiently collected (Supplementary Figure S1B). Through rarefaction and species accumulation curves, we also found that the species richness of the control group was the highest, followed by that of the PPI and UC groups. The rank-abundance curves covers both the uniformity and richness of the species in a sample. In the present study, the curves for the three groups were flat, indicating a more uniform species distribution. In addition, the curves for the control group had the largest range span on the horizontal axis, indicating the highest species abundance, whereas the UC group had the smallest span and lowest species abundance (Supplementary Figure S1C).

#### 3.3.2 OTU cluster analysis

The OTU annotation at different taxonomic levels (superkingdom, phylum, class, order, family, genus, and species) for each sample are presented in Supplementary Table S1. A total of 3,551 OTUs were obtained from the 20 samples, including 1,148 from the control group, 930 from the PPI group, and 1,473 from the UC group. A Venn diagram shows that the three groups shared 222 OTUs, whereas the unique OTUs of each group were 583, 477, and 999, respectively (Supplementary Figure S1D). Indicator analysis showed that the top 100 OTUs had the highest relative abundances (FDR-corrected *p* < 0.05; Supplementary Figure S1E).

#### 3.3.3 Alpha and beta diversity analysis

**Figure 3:**
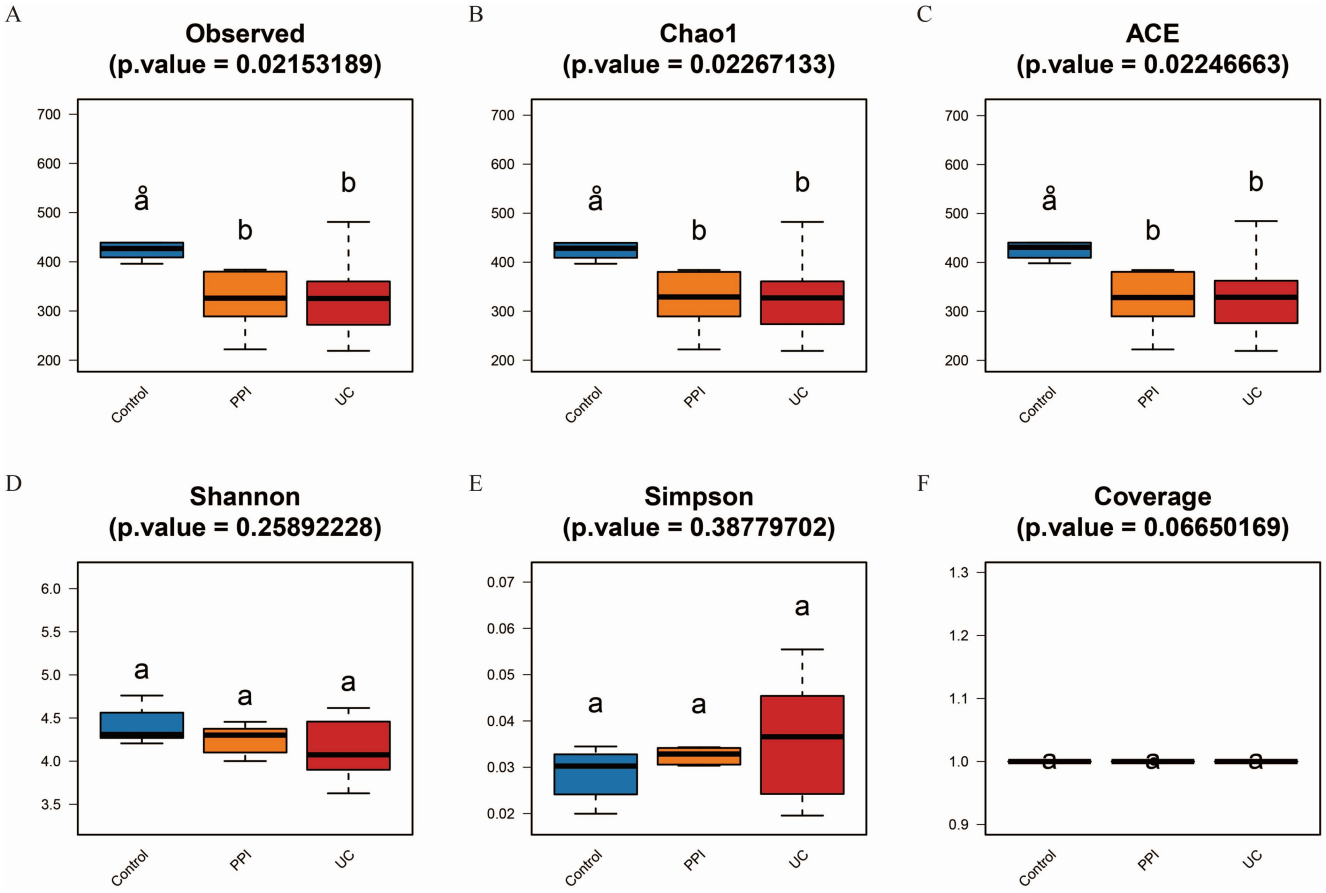
Alpha diversity analysis among control (*n* = 5), PPI (*n* = 5) and UC (*n* = 10) groups. **(A)** Observed_species index. **(B)** Chao1 index. **(C)** ACE index. **(D)** Shannon index. **(E)** Simpson index. **(F)** Coverage index. All the data in this section follow a normal distribution. Based on one-way ANOVA test, *p* < 0.05 indicates significant difference among groups. The horizontal axis represents the different groups, and the vertical axis represents the diversity index value. Each color represents a group: blue for control, orange for PPI, and red for UC group.

Alpha diversity is the analysis of species diversity in samples based on OTU species and abundance. Observed_species, Chao1, and ACE indices are mainly used to calculate community richness and are positively correlated with it. Similarly, Shannon, Simpson and coverage indices are used to calculate community diversity. The Shannon and coverage indices are positively correlated with community diversity, whereas the Simpson index is negatively correlated. Based on the one-way ANOVA test, the alpha diversity analysis showed that the Observed_species, Chao1, ACE, Shannon, and coverage indices were lower in the PPI and UC groups than in the control group, whereas the Simpson index trended in the opposite direction. These results suggested that the richness and gut microbiota diversity in mice decreased after induction with OME and DSS (Figure 3).

**Figure 4:**
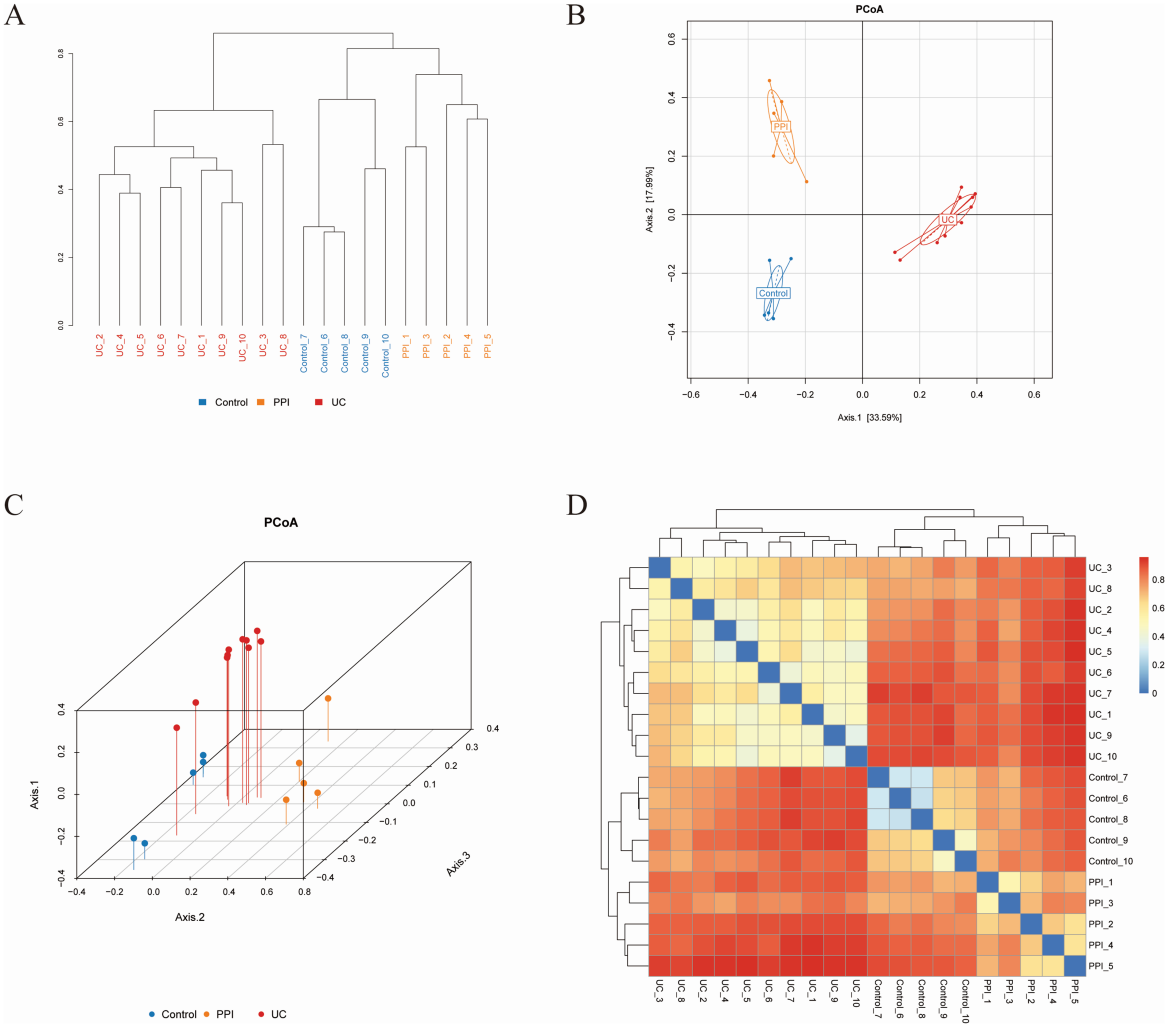
Beta diversity analysis among control (*n* = 5), PPI (*n* = 5) and UC (*n* = 10) groups based on Bray-Curtis distance. **(A)** The clustering tree. The length of the branches represents the distance between the samples, and the closer the branches are, the more similar the species composition of the samples. **(B)** Two-dimensional diagram of principal coordinates analysis (PCoA) based on OTU abundance. **(C)** Three-dimensional diagram of PCoA based on OTU abundance. **(D)** Heatmap of PCoA based on OTU abundance. Each dot represents a sample, and the closer the dots are, the more similar the samples. Each color represents a group: blue for control group, orange for PPI group, and red for UC group.

A sample clustering tree can describe and compare similarities and differences in species among groups using a general view-based approach. The more similar the samples are, the higher the clustering priority. The clustering tree among the UC, PPI, and control groups showed that the similarity in species composition was high among all samples in the intra-group, while there were significant differences in the inter-group (Figure 4A). PCoA showed the relationship between the species composition of each group in two-dimensional coordinates. In the coordinate plots, the closer the distance between the samples, the more similar is the species composition, according to which differences in species composition and structure can be observed. Based on Bray-Curtis, a significant separation in gut microbiota composition among the control, PPI, and UC groups was revealed based on 2D and 3D images (Figures 4B–D). Similarly, principal component analysis and non-metric multidimensional scaling also revealed distinct distribution patterns of the gut microbiota in distinct groups of mice (Supplementary Figures S2A–D). In addition, analysis of similarities based on the R language vegan package anosim function showed that the inter-group differences in the control, PPI, and UC groups were significantly larger than the intra-group differences (Supplementary Table S2, *p* < 0.001). These results showed that the similarity in species composition was high among all samples in the intra-group, whereas there were significant differences in the inter-group among the UC, PPI, and control groups.

#### 3.3.4 Gut microbiota structural analysis

We analyzed the gut microbiota structure of the control, PPI, and UC groups at six levels: phylum, class, order, family, genus, and species. Multilayer analysis of the gut microbiota revealed significant differences in relative microbial abundance among the three groups. At the phylum level, the dominant bacteria in the three groups were *Bacteroidetes*, *Firmicutes*, and *Proteobacteria*, but their proportions differed (control: 49.59, 38.09, and 9.98%; PPI: 41.51, 50.03, and 4.8%; UC: 39.33, 38.96, and 19.26%, respectively). The other dominant bacteria in the control, PPI, and UC groups were *Actinobacteria* (1.61%), *Verrucomicrobia* (1.23%), and *Deferribacteres* (1.24%) (Supplementary Figure S3).

At the class level, the five most dominant bacteria in the control group were *Bacteroidia* (49.58%), *Clostridia* (17.54%), *Erysipelotrichia* (14.56%), *Bacilli* (5.58%), and *Epsilonproteobacteria* (4.8%). The PPI group included *Bacteroidia* (41.51%), *Clostridia* (25.63%), *Bacilli* (21.18%), *Erysipelotrichia* (3.07%), and *Alphaproteobacteria* (1.52%). In the UC group, *Bacteroidia* (39.33%), *Clostridia* (23.56%), *Bacilli* (11.42%), *Epsilonproteobacteria* (6.34%), and *Gammaproteobacteria* (6.25%) were detected. Compared with that in the control group, *Bacteroidia* and *Erysipelotrichia* abundance decreased in the PPI and UC groups, whereas *Clostridia* and *Bacilli* showed the opposite (Supplementary Figure S4).

At the order level, the top five dominant bacteria in the control group included *Bacteroidales* (49.58%), *Clostridiales* (17.42%), *Erysipelotrichales* (14.56%), *Lactobacillales* (5.57%), *Campylobacterales* (4.8%); in the PPI group included *Bacteroidales* (41.51%), *Clostridiales* (25.37%), *Lactobacillales* (21.14%), *Erysipelotrichales* (3.07%), and *Campylobacterales* (1.33%); and in the UC group included *Bacteroidales* (39.33%), *Clostridiales* (23.45%), *Lactobacillales* (11.27%), *Campylobacterales* (6.34%), and *Enterobacteriales* (6.02%). *Bacteroidales* and *Erysipelotrichales* abundance decreased in the PPI and UC groups, whereas *Clostridiales* and *Lactobacillales* showed the opposite trend (Supplementary Figure S5).

At the family level, the top five dominant bacteria in the control group included *Porphyromonadaceae* (39.01%), *Erysipelotrichaceae* (14.56%), *Lachnospiraceae* (10.11%), *Ruminococcaceae* (6.03%), and *Lactobacillaceae* (5.53%); in the PPI group included *Porphyromonadaceae* (28.81%), *Lactobacillaceae* (17.81%), *Lachnospiraceae* (16.84%), *Prevotellaceae* (8.18%), and *Ruminococcaceae* (7.23%); and in the UC group included *Porphyromonadaceae* (19.11%), *Bacteroidaceae* (17.04%), *Lachnospiraceae* (11.57%), *Lactobacillaceae* (11%), and *Ruminococcaceae* (6.95%). *Porphyromonadaceae* and *Erysipelotrichaceae* abundance decreased in the PPI and UC groups, whereas *Lachnospiraceae* and *Lactobacillaceae* showed the opposite trend (Supplementary Figure S6).

At the genus level, the top five dominant bacteria in the control group included *Allobaculum* (10.21%), *Lactobacillus* (5.53%), *Helicobacter* (4.8%), *Bacteroides* (3.37%), and *Alloprevotella* (2.35%); in the PPI group included *Lactobacillus* (17.81%), *Eisenbergiella* (4.61%), *Prevotella* (4.27%), *Bacteroides* (3.41%), and *Streptococcus* (3.3%); and in UC group included *Bacteroides* (17.04%), *Lactobacillus* (11%), *Helicobacter* (6.34%), *Escherichia/Shigella* (5.98%), and *Parabacteroides* (5.9%) (Supplementary Figure S7).

Finally, at the species level, there was a significant difference in the gut microbiota of each group with no obvious correlation. *Streptococcus hyointestinalis* (3.28%) was also observed in the PPI group (Supplementary Figure S8).

#### 3.3.5 Differential abundance analysis

**Figure 5:**
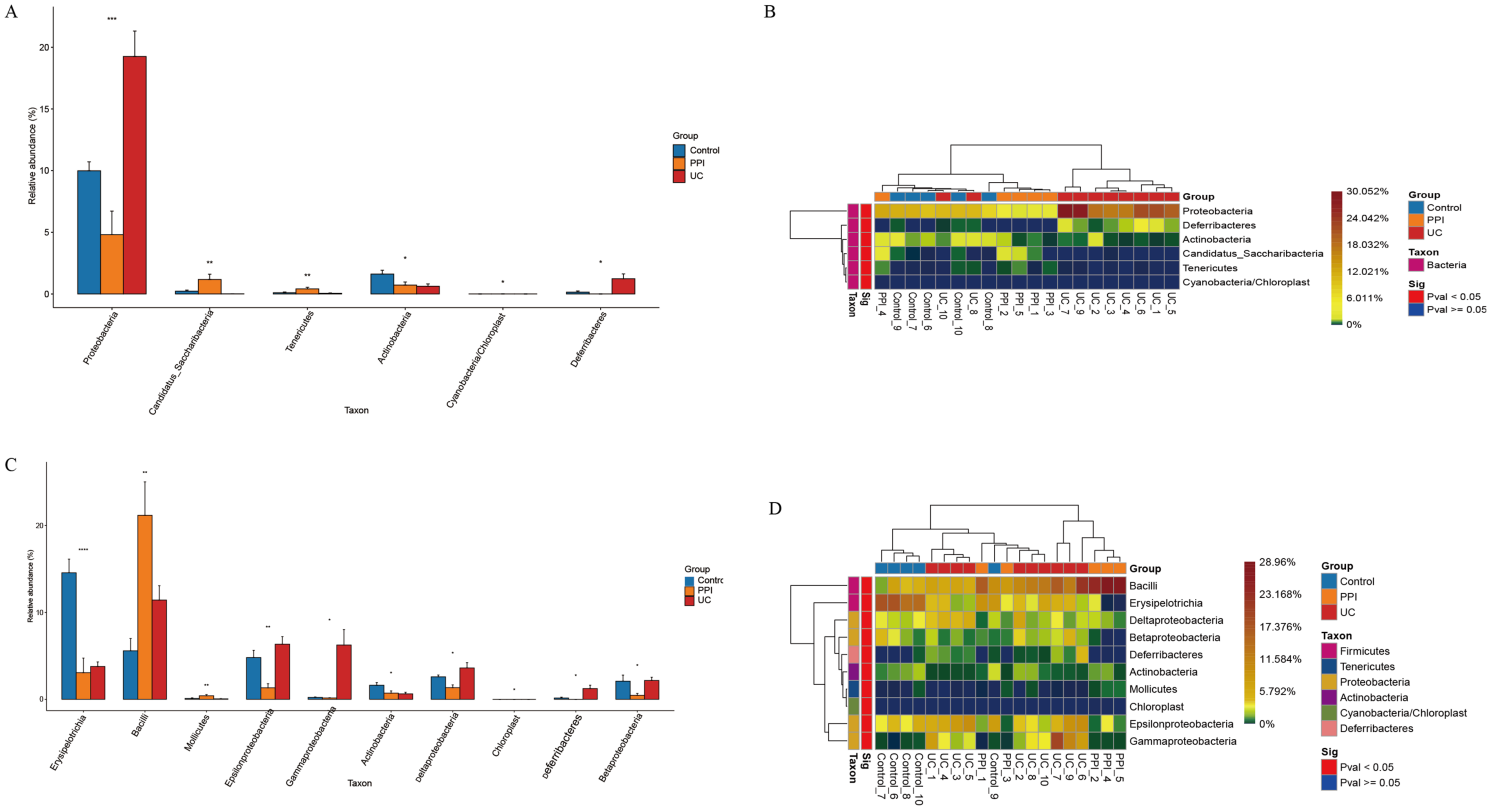
Differences in composition of gut microbiota among control (*n* = 5), PPI (*n* = 5), and UC (*n* = 10) groups. **(A)** Barplot of differential microbial taxa at the phylum level. **(B)** Heatmap of differential microbial taxa at the phylum level. **(C)** Barplot of differential microbial taxa at the class level. **(D)** Heatmap of differential microbial taxa at the class level. Based on ANOVA, *p* < 0.05 indicates significant difference among groups. Barplot: Horizontal axis represents the different groups, and the vertical axis represents the relative abundance value of species. *Indicates 0.01 ≤ *p* < 0.05, **indicates 0.001 ≤ *p* < 0.01, ***indicates 0.0001 ≤ *p* < 0.001, and ****indicates *p* < 0.0001. Heatmap: Horizontal axis represents the different groups, the vertical axis represents the different species, and the color gradient from blue to red indicates the relative abundance of species from small to large.

ANOVA was used to analyze differences in abundance among the three groups in terms of phylum, class, order, family, genus, and species. The UC group demonstrated significant enrichment of *Proteobacteria* and *Deferribacteres* at the phylum level, whereas *Candidatus*, *Saccharibacteria* and *Tenericutes* were dominant in the PPI group. Compared with that in the control group, the enrichment of *Actinobacteria* was reduced in the PPI and UC groups (Figures 5A,B). At the class level, the PPI and UC groups exhibited a notable increase in the abundance of *Bacilli*, reflecting the potential enrichment of the inflammation-associated microbiota. In contrast, *Erysipelotrichia* and *Actinobacteria* decreased with the same trend in both groups, suggesting that OME and DSS usage may alter the ecological dominance of specific microbial populations (Figures 5C,D). In addition, the microbial taxa with increased abundance in the PPI and UC groups also included *Lactobacillales*, *Bacteroidaceae*, *Lactobacillaceae*, *Bacteroides*, *Lactobacillus*, *Erysipelotrichaceae_incertae_sedis*, *Escherichia/Shigella*, *Clostridium_XlVa*, *Blautia*, *Enterococcus*, *Parabacteroides_distasonis* and *Parabacteroides_gordonii*, while the microbial taxa with decreased abundance also included *Erysipelotrichales*, *Coriobacteriales*, *Erysipelotrichaceae*, *Coriobacteriaceae*, *Porphyromonadaceae*, *Allobaculum*, *Anaerobacterium*, *Intestinimonas*, and *Lactobacillus_intestinalis* compared with that in the control group in order, family, genus, and species levels, respectively (Supplementary Figures S9–S12).

#### 3.3.6 Key differential microbial taxa

**Figure 6:**
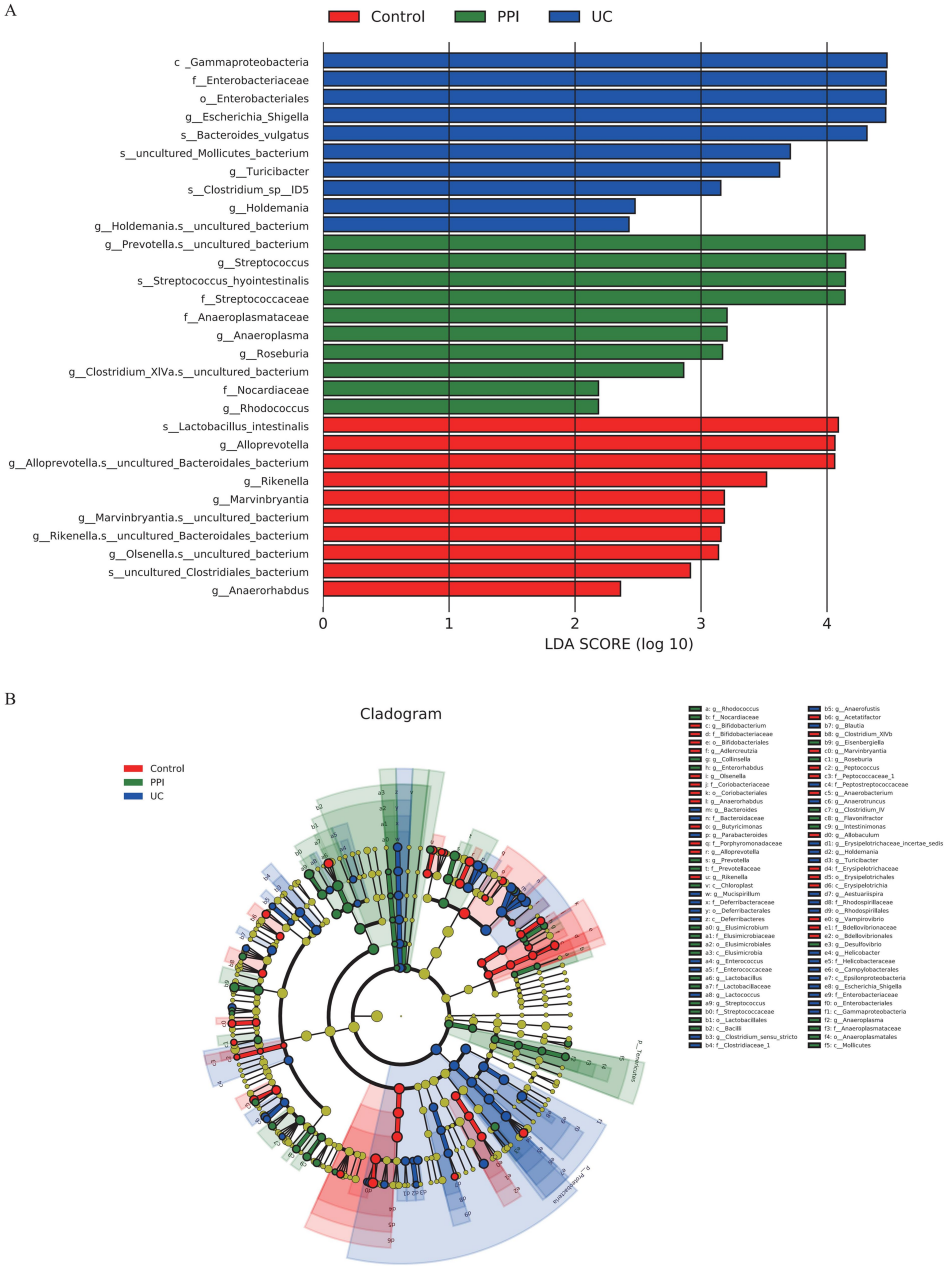
Intergroup differences in gut microbiota among control (*n* = 5), PPI (*n* = 5) and UC (*n* = 10) groups detected by LEfSe analysis. **(A)** Linear discriminant analysis (LDA) score distribution. Defined |LDA| > 2 and *p* < 0.05 of LDA scores indicates significant difference among groups. **(B)** A cladogram shows the taxonomic structure and the relative abundance of the identified taxa. The size of each dot is proportional to the relative abundance of each taxon.

The linear discriminant analysis effect size (LEfSe) tool was used to identify the microbial taxa most likely to explain the intergroup differences. The results showed that the UC group had the highest linear discriminant analysis (LDA) scores for *Gammaproteobacteria*, *Enterobacteriaceae*, and *Escherichia/Shigella*, indicating that these taxa may serve as key microbial biomarkers associated with UC because of their significant intergroup differences. In the PPI group, *Prevotella.s_*uncultured_bacterium and *Streptococcus* exhibited the highest LDA scores, suggesting that PPI usage might selectively amplify these taxa, thereby influencing gut microbiota ecology (Figure 6A). Furthermore, a cladogram visually illustrated the taxonomic distribution patterns of key taxa enriched in the different groups. The enriched taxa in the UC group were primarily clustered under the phyla *Proteobacteria* and *Bacteroidetes*, which are commonly associated with inflammatory conditions in the gut. In contrast, the control group was predominantly enriched in taxa from the *phylum Firmicutes*. Similarly, the differential taxa in the PPI group were also concentrated within the *phylum Firmicutes* but were more specifically associated with lactic acid bacteria (Figure 6B).

#### 3.3.7 Functional annotation and clustering analysis

Functional annotation and classification of samples from the control, PPI, and UC groups revealed significant differences in functional distribution and relative abundance among the experimental groups. In the Clusters of Orthologous Groups of proteins (COG) functional annotations, the functions related to “translation, ribosomal structure and biogenesis” and “amino acid transport and metabolism” were the most abundant across all groups. This indicates that the gut microbiota primarily centers on protein synthesis, ribosomal functionality, and amino acid metabolism, whereas a substantial proportion of annotated functions remain unknown (Supplementary Figures S13A–C). However, in the PPI group, functions related to “cell wall/membrane/envelope biogenesis” and “nucleotide transport and metabolism” also showed relatively high enrichment, suggesting that PPI usage may influence gut defense mechanisms and nucleotide metabolism. Additionally, an increase in “signal transduction mechanisms” implies that PPI might impact microecological functionality by modulating signaling pathways (Supplementary Figure S13B). These trends were even more pronounced in the UC group. Moreover, significant enhancements in “energy production and conversion” and “carbohydrate transport and metabolism” functions in the UC group suggest that the gut microbiota in UC patients may adapt to the inflammatory environment by boosting energy metabolism. Furthermore, increased functions related to “replication, recombination and repair” and “mobilome: prophages, transposons” may indicate microbial instability and heightened horizontal gene transfer within the microbiota (Supplementary Figure S13C).

Further heatmap and clustering analyses revealed differences and similarities in the top 30 functions with the highest relative abundances across the groups. The enrichment of functions annotated by COGs, the Kyoto Encyclopedia of Genes and Genomes (KEGG), and KEGG Orthology (KO) collectively reflected the adaptive regulatory responses of the gut microbiota to environmental pressures, such as inflammatory conditions or drug interventions (Supplementary Figures S14A–C). For example, the enrichment of glycosyltransferases involved in cell wall biosynthesis in the COG indicates active microbial metabolism in maintaining cell wall structure and stability. Similarly, the annotation of “biosynthesis of ansamycins” in KEGG suggests that microbes may produce secondary metabolites with antibacterial properties to compete ecologically. In contrast to these shared functional annotations, the metabolic pathway database MetaCyc revealed distinct functional enrichment among the groups. In the control group, functions were predominantly enriched in pathways such as “L-lysine biosynthesis III” and “UMP biosynthesis,” indicating a focus on amino acid and nucleotide metabolism under healthy conditions. The PPI group exhibited enrichment in “guanosine deoxyribonucleotides *de novo* biosynthesis II” and “adenosine deoxyribonucleotides de novo biosynthesis II,” suggesting that PPI usage may enhance nucleotide metabolism pathways. Meanwhile, the UC group showed enrichment in pathways such as “gondoate biosynthesis (anaerobic)” and “pyruvate fermentation to isobutanol (engineered),” which may be associated with specific metabolic demands in inflammatory environments, reflecting microbial metabolic adaptability to these conditions (Supplementary Figure S14D).

## 4 Discussion

This study was designed as an exploratory animal experiment focusing on the effects of chronic PPI exposure on gut microbiota and inflammatory readouts, rather than establishing strict clinical dose equivalence. In this work, we investigated the impact of long-term PPI and DSS exposure on the gut microbiota. The results demonstrated that both DSS and prolonged PPI use significantly reduced the richness and diversity of the gut microbiota. Specifically, there was a notable decrease in beneficial bacteria such as *Faecalibacterium*, alongside a significant increase in potentially pathogenic taxa such as *Escherichia/Shigella* and *Enterococcaceae*. Furthermore, functional enrichment analyses suggested that PPIs may disrupt the microbial ecological balance by affecting the gut defense mechanisms and nucleotide metabolism. These findings provide important insights into the mechanisms by which PPIs induce the gut microbiota and immune dysregulation. They also offer new perspectives and experimental evidence regarding the pathogenesis of PPI-mediated UC.

Previous studies have demonstrated that *C. difficile* is a potential risk factor for adverse outcomes, such as gut infections, small gut bacterial overgrowth, spontaneous bacterial peritonitis, and IBD. The use of PPIs significantly increases the abundance of *Enterococcaceae*, a risk factor for *C. difficile* infection (Hopkins et al., 2022; Naito et al., 2018; Carr, 2018; Martinez et al., 2022). This finding aligns with the results of our study, which showed significant upregulation of *Enterococcus* and *Enterococcaceae* in the gut microbiota of UC and PPI-treated mice. The pathogenic potential of enterococci has become increasingly recognized in recent years, particularly for multidrug-resistant strains of *Enterococcus faecium* (*E. faecium*), which have been implicated in various infections. Barnett et al. (2010) and Li et al. (2024) reported that the inoculation of *Enterococcus* in IL-10^−/−^ mice exacerbated colonic inflammation. Sequencing analyses further revealed that *Enterococcus*-induced colitis closely resembled the changes in gene expression observed in human IBD. Similarly, Seishima et al. (2019) conducted whole-genome shotgun sequencing of stool samples from patients with UC and healthy individuals and identified *E. faecium* as the most differentially abundant species between the two groups. Moreover, fecal transplants, *E. faecium* isolates from UC patients, and exogenous inflammatory *E. faecium* strains (ATCC 19434) promoted pathological inflammation and upregulated inflammatory cytokine expression in the colons of IL-10^−/−^ mice (Steck et al., 2011; Ocvirk et al., 2015). These findings suggest that *E. faecium* within enterococci may be an important contributor to UC pathogenesis and may serve as a key bacterial mediator in PPI-induced UC. However, the precise molecular mechanisms remain unclear and require further investigation.

Functional enrichment analysis and previous studies indicate that *E.faecium*, *Escherichia/Shigella*, and other Gammaproteobacteria possess unique peptidoglycan structures in their cell walls (Griffin et al., 2021; Ago et al., 2023). These microbes undergo extensive peptidoglycan remodeling and turnover, generating abundant, smaller, non-crosslinked fragments that play essential roles in host defense against intestinal pathogens (Rangan et al., 2016; Tian and Han, 2022; Abramov et al., 2023). For example, peptidoglycan recognition proteins (PGLYRPs) have been shown to be significantly associated with UC, suggesting a potential role in IBD pathogenesis (Zulfiqar et al., 2013). Peptidoglycan remodeling is also closely linked to the activity of secreted antigen A (SagA) in *E. faecium* (Kim et al., 2019; Teng et al., 2003). SagA, a peptidoglycan hydrolase containing an NlpC/p60 domain, preferentially hydrolyzes cross-linked Lys-type peptidoglycan fragments and generates immunologically active non-crosslinked fragments such as muramyl dipeptide (MDP) and GlcNAc-MDP (Rangan et al., 2016; Espinosa et al., 2020; Kim et al., 2020). MDP and GlcNAc-MDP are classical ligands and intracellular sensor of the NOD2 receptor (Gao et al., 2022; Okai et al., 2024). Previous studies have shown that gram-positive bacteria promote the occurrence of colitis by upregulating the MDP-NOD2 pathway (Luo et al., 2021). In the Portuguese population, NOD2 mutations do not increase the risk of UC but are associated with a more aggressive course (Freire et al., 2014). In parallel, *Escherichia/Shigella* are characterized by virulence factors such as Shiga toxin, lipopolysaccharide (LPS), and adhesins, which disrupt epithelial integrity and promote bacterial translocation, thereby contributing to immune activation and inflammation in UC (O'Brien et al., 1984; Lu et al., 2025).

This study had several limitations that warrant discussion. First, it was based solely on fecal samples from 20 mice, representing a relatively small sample size. This limitation may not adequately capture the heterogeneity of gut microbiota within the population, potentially affecting the generalizability of our conclusions. Second, the study inferred the relationships between the microbiota, host immunity, and intestinal diseases through microbial abundance and functional enrichment analyses of fecal samples. However, these studies did not incorporate direct measurements of host immune parameters or histological analyses of the intestinal tissues, making it challenging to establish causal relationships between microbial changes and inflammatory states. Third, this study focused exclusively on changes in gut microbiota in fecal samples without considering microbial variations in other regions of the gastrointestinal tract, such as the small intestine or colon, and their potential roles in PPI exposure and UC pathogenesis. In addition, although our findings suggest a potential involvement of the NOD2–NLRP3 inflammasome pathway, we did not perform direct molecular or functional validation, and this remains an important direction for future research. Finally, as an exploratory study, our work was primarily intended to provide preliminary laboratory evidence on the effects of chronic PPI exposure on gut microbiota and intestinal inflammation. While the findings complement existing epidemiological observations, they should not be interpreted as definitive causal conclusions. Regarding dose selection, although the chosen omeprazole regimen is clinically plausible based on human-equivalent scaling, it was not directly validated through pharmacokinetic or exposure–response studies and therefore may not fully reflect human outcomes.

## 5 Conclusion

This study revealed the significant impact of long-term PPI exposure on the gut microbiota. The findings demonstrated that both PPIs and DSS significantly reduced the diversity and richness of the gut microbiota while promoting the enrichment of pro-inflammatory taxa, such as *Enterococcaceae* and *Escherichia_Shigella*. While these results provide preliminary insights into the potential role of PPIs in gut microbiota dysbiosis and the pathogenesis of UC, further validation is warranted. Future studies might consider integrating host immune profiling, histological assessment, and multi-omics approaches to strengthen the understanding of microbiota–host interactions and to inform the development of more targeted intervention strategies.

## Data Availability

The raw sequence data reported in this paper have been deposited in the Genome Sequence Archive (Genomics, Proteomics and Bioinformatics 2021) in National Genomics Data Center (Nucleic Acids Res 2022), China National Center for Bioinformation/Beijing Institute of Genomics, Chinese Academy of Sciences (GSA: CRA021599) that are publicly accessible at https://ngdc.cncb.ac.cn/gsa.

## Footnotes

^1^ http://drive5.com/uchime/gold.fa

## Supplementary material

SUPPLEMENTARY FIGURE S1 Sequencing depth and species diversity. **(A)** Rarefaction curve. The horizontal axis represents the number of sequences randomly selected from the sample, and the vertical axis represents the number of OTUs/ASVs to which the sequence belongs. Each curve represents a sample, and different colors represent different samples or groups. **(B)** Species accumulation curves. The horizontal axis represents the number of random samples, and the vertical axis represents the total number of OTUs included in the sampled samples. **(C)** Rank-abundance curves. The horizontal axis represents OTUs ranked from most to least in terms of the number of sequences it contains. For example, “500” represents OTUs with the 500th abundance in the sample. The vertical axis represents the relative abundance of each OTU. Each curve represents a sample, and different colors represent different groups. **(D)** Venn diagram of OUTs. Different groups are represented by different colors, and the overlapping areas of different color circles indicate the number of common species. **(E)** Indicator analysis (the top 100 OTUs with the highest relative abundance, FDR-corrected *p* < 0.05). The vertical axis represents OTUs, the horizontal axis represents the different groups, and the bubble size represents the indicator value of each species.

SUPPLEMENTARY FIGURE S2 Beta diversity analysis among control (*n* = 5), PPI (*n* = 5), and UC (*n* = 10) groups based on Bray-Curtis distance. **(A)** Two-dimensional diagram of principal component analysis (PCA) based on OTU abundance. **(B)** Three-dimensional diagram of PCA based on OTU abundance. **(C)** Two-dimensional diagram of nonmetric multidimensional scaling (NMDS) based on OTU abundance. **(D)** Three-dimensional diagram of NMDS based on OTU abundance. Each dot represents a sample, and each color represents a group: blue for control, orange for PPI, and red for UC.

SUPPLEMENTARY FIGURE S3 Structure analysis of gut microbiota among control (*n* = 5), PPI (*n* = 5), and UC (*n* = 10) groups at the phylum level. **(A)** Barplot of species composition. **(B)** Bubble plot of abundance distribution. **(C)** Heatmap of sample clustering. **(D)** Proportions of main bacteria of the control group. **(E)** Proportions of main bacteria of the PPI group. **(F)** Proportions of main bacteria of the UC group. Barplot: Each bar represents a group; the vertical axis represents the relative abundance value. The average relative abundance of all species in each group adds up to 1, and each color corresponds to one species. Bubble: The horizontal axis represents the groups, the vertical axis represents the high-abundance species, and the size of the dots represents the relative abundance of the species in the sample. Heatmap: The horizontal axis represents the sample, the vertical axis represents the top 100 species with the highest abundance at the taxonomic level, and the color gradient from blue to red indicates the species abundance from small to large. Pie plot: Different colors represent different species, and the larger the fan area, the higher the abundance of that species.

SUPPLEMENTARY FIGURE S4 Structure analysis of gut microbiota among control (*n* = 5), PPI (*n* = 5), and UC (*n* = 10) groups at the class level. **(A)** Barplot of species composition. **(B)** Bubble plot of abundance distribution. **(C)** Heatmap of sample clustering. **(D)** Proportions of main bacteria of the control group. **(E)** Proportions of main bacteria of the PPI group. **(F)** Proportions of main bacteria of the UC group.

SUPPLEMENTARY FIGURE S5 Structure analysis of gut microbiota among control (*n* = 5), PPI (*n* = 5) and UC (*n* = 10) groups at the order level. **(A)** Barplot of species composition. **(B)** Bubble plot of abundance distribution. **(C)** Heatmap of sample clustering. **(D)** Proportions of main bacteria of the control group. **(E)** Proportions of main bacteria of the PPI group. **(F)** Proportions of main bacteria of the UC group.

SUPPLEMENTARY FIGURE S6 Structure analysis of gut microbiota among control (*n* = 5), PPI (*n* = 5), and UC (*n* = 10) groups at the family level. **(A)** Barplot of species composition. **(B)** Bubble plot of abundance distribution. **(C)** Heatmap of sample clustering. **(D)** Proportions of main bacteria of the control group. (**E**) Proportions of main bacteria of the PPI group. **(F)** Proportions of main bacteria of the UC group.

SUPPLEMENTARY FIGURE S7 Structure analysis of gut microbiota among control (*n* = 5), PPI (*n* = 5), and UC (*n* = 10) groups at the genus level. **(A)** Barplot of species composition. **(B)** Bubble plot of abundance distribution. **(C)** Heatmap of sample clustering. **(D)** Proportions of main bacteria of the control group. **(E)** Proportions of main bacteria of the PPI group. **(F)** Proportions of main bacteria of the UC group.

SUPPLEMENTARY FIGURE S8 Structure analysis of gut microbiota among control (*n* = 5), PPI (*n* = 5), and UC (*n* = 10) groups at the species level. **(A)** Barplot of species composition. **(B)** Bubble plot of abundance distribution. **(C)** Heatmap of sample clustering. **(D)** Proportions of main bacteria of the control group. **(E)** Proportions of main bacteria of the PPI group. **(F)** Proportions of main bacteria of the UC group.

SUPPLEMENTARY FIGURE S9 Difference in composition of gut microbiota among control (*n* = 5), PPI (*n* = 5), and UC (*n* = 10) groups. **(A)** Barplot of differential microbial taxa at the order level. **(B)** Heatmap of differential microbial taxa at the order level.

SUPPLEMENTARY FIGURE S10 Difference in composition of gut microbiota among control (*n* = 5), PPI (*n* = 5), and UC (*n* = 10) groups. **(A)** Barplot of differential microbial taxa at the family level. **(B)** Heatmap of differential microbial taxa at the family level.

SUPPLEMENTARY FIGURE S11 Difference in composition of gut microbiota among control (*n* = 5), PPI (*n* = 5), and UC (*n* = 10) groups. **(A)** Barplot of differential microbial taxa at the genus level. **(B)** Heatmap of differential microbial taxa at the genus level.

SUPPLEMENTARY FIGURE S12 Difference in composition of gut microbiota among control (*n* = 5), PPI (*n* = 5), and UC (*n* = 10) groups. **(A)** Barplot of differential microbial taxa at the species level. **(B)** Heatmap of differential microbial taxa at the species level.

SUPPLEMENTARY FIGURE S13 Functional annotation and classification of samples from the control (*n* = 5), PPI (*n* = 5), and UC (*n* = 10) groups. **(A)** Control group. **(B)** PPI group. (**C**) UC group. The horizontal axis represents the Clusters of Orthologous Groups (COG) function, and the vertical axis represents the COG function abundance.

SUPPLEMENTARY FIGURE S14 Heatmap and clustering analyses of differences and similarities in the top 30 functions with the highest relative abundances among control (*n* = 5), PPI (*n* = 5), and UC (*n* = 10) groups. **(A)** Enrichment of functions annotated by Clusters of Orthologous Groups of proteins (COG). **(B)** Enrichment of functions annotated by clusters of Kyoto Encyclopedia of Genes and Genomes (KEGG). **(C)** Enrichment of functions annotated by clusters of KEGG Orthology (KO). **(D)** Enrichment of functions annotated by clusters of metabolic pathway database (MetaCyc). The horizontal axis represents the different sample, the vertical axis represents the top 30 functions with the highest abundance, and the gradient color from light to dark indicates the relative abundance of the functions from small to large.
