# Supplementary figures and images for: Proton pump inhibitors and gut microbiota dysbiosis: insights into the pathogenesis of ulcerative colitis
Source: Front Microbiol. 2025 Oct 30;16:1657865. doi: 10.3389/fmicb.2025.1657865 (PMC12612687; doi:10.3389/fmicb.2025.1657865)

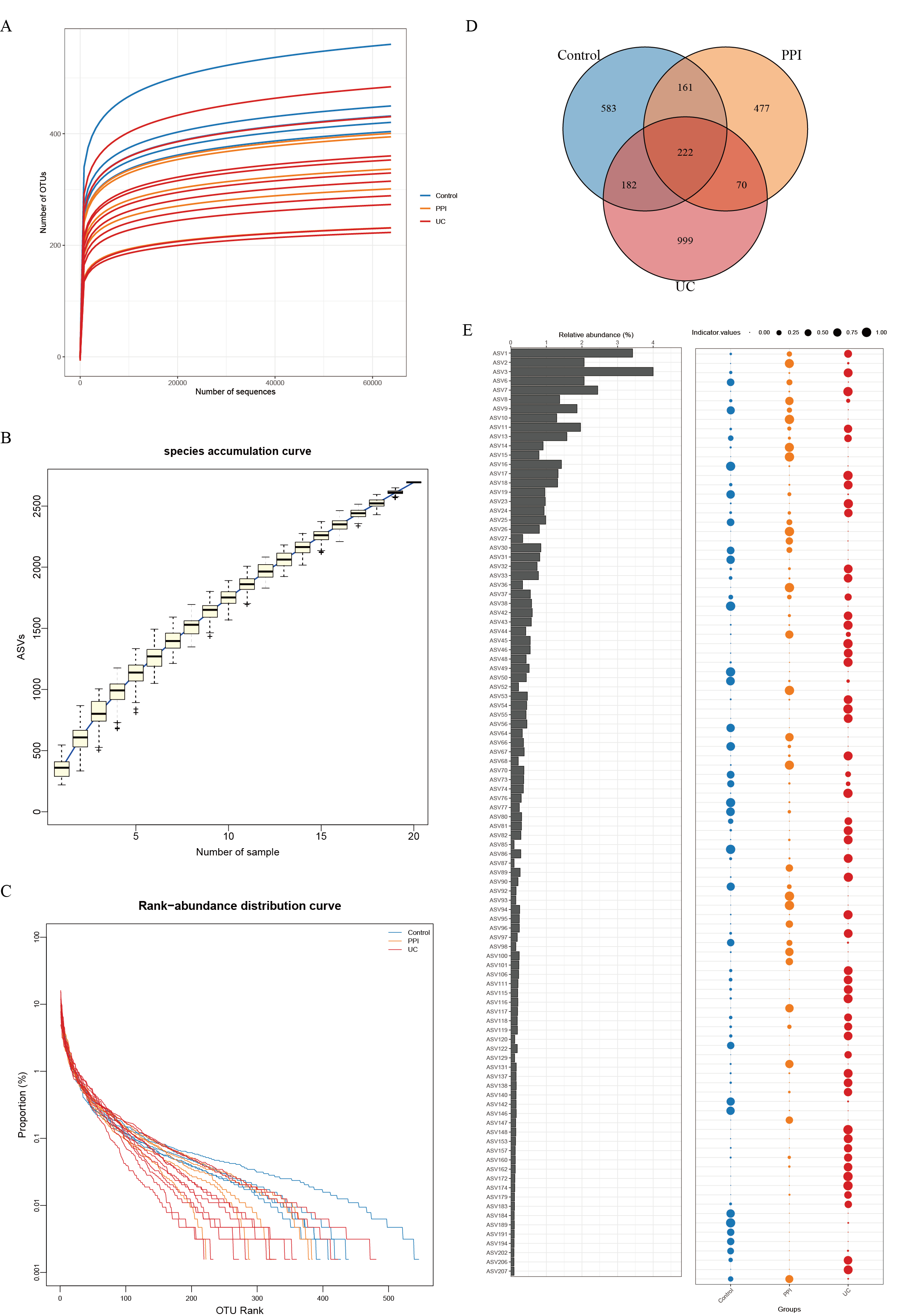

Supplement: SUPPLEMENTARY FIGURE S1 — Sequencing depth and species diversity. (A) Rarefaction curve. The horizontal axis represents the number of sequences randomly selected from the sample, and the vertical axis represents the number of OTUs/ASVs to which the sequence belongs. Each curve represents a sample, and different colors represent different samples or groups. (B) Species accumulation curves. The horizontal axis represents the number of random samples, and the vertical axis represents the total number of OTUs included in the sampled samples. (C) Rank-abundance curves. The horizontal axis represents OTUs ranked from most to least in terms of the number of sequences it contains. For example, “500” represents OTUs with the 500th abundance in the sample. The vertical axis represents the relative abundance of each OTU. Each curve represents a sample, and different colors represent different groups. (D) Venn diagram of OUTs. Different groups are represented by different colors, and the overlapping areas of different color circles indicate the number of common species. (E) Indicator analysis (the top 100 OTUs with the highest relative abundance, FDR-corrected p < 0.05). The vertical axis represents OTUs, the horizontal axis represents the different groups, and the bubble size represents the indicator value of each species. [file Image_1.jpeg]

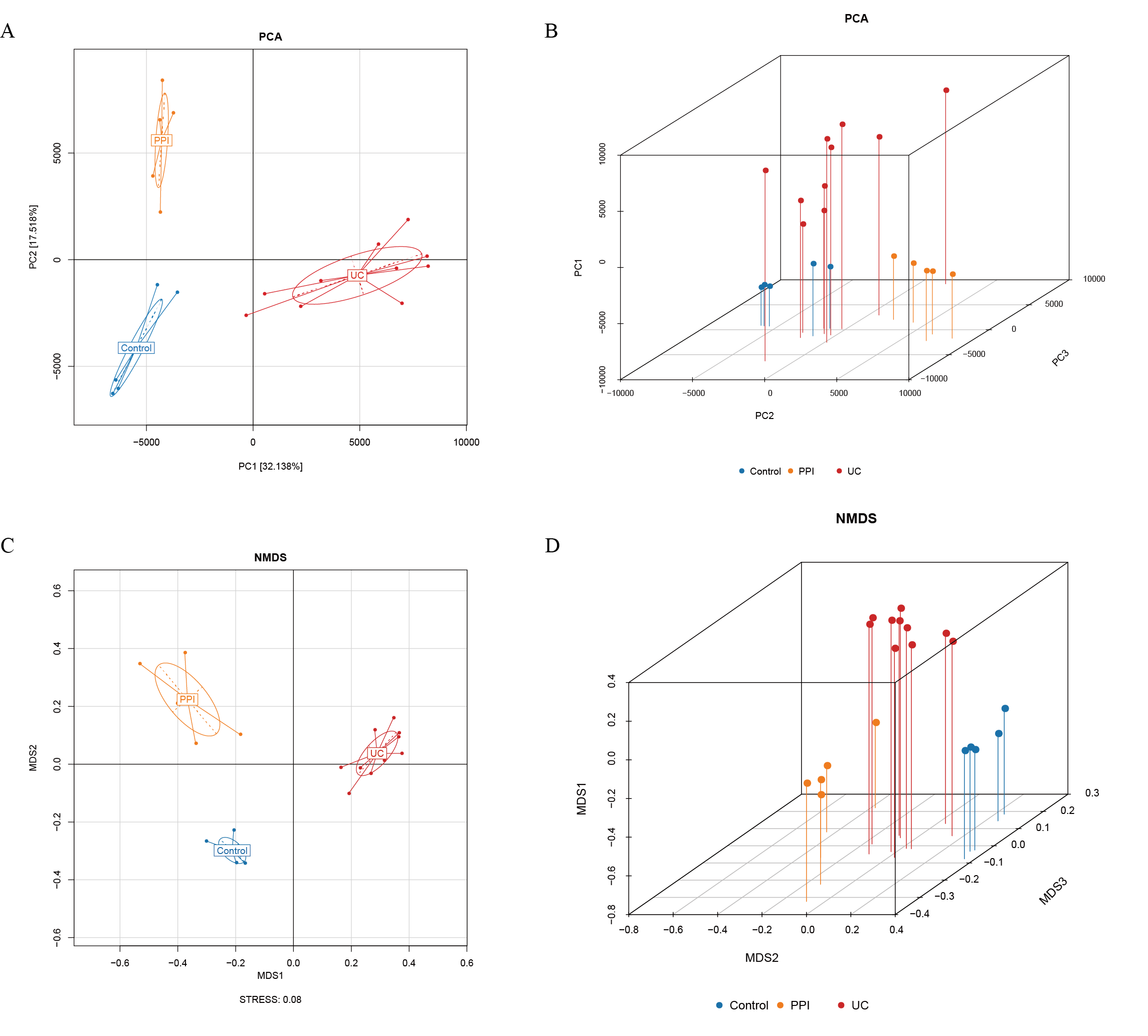

Supplement: SUPPLEMENTARY FIGURE S2 — Beta diversity analysis among control (n = 5), PPI (n = 5), and UC (n = 10) groups based on Bray-Curtis distance. (A) Two-dimensional diagram of principal component analysis (PCA) based on OTU abundance. (B) Three-dimensional diagram of PCA based on OTU abundance. (C) Two-dimensional diagram of nonmetric multidimensional scaling (NMDS) based on OTU abundance. (D) Three-dimensional diagram of NMDS based on OTU abundance. Each dot represents a sample, and each color represents a group: blue for control, orange for PPI, and red for UC. [file Image_2.jpeg]

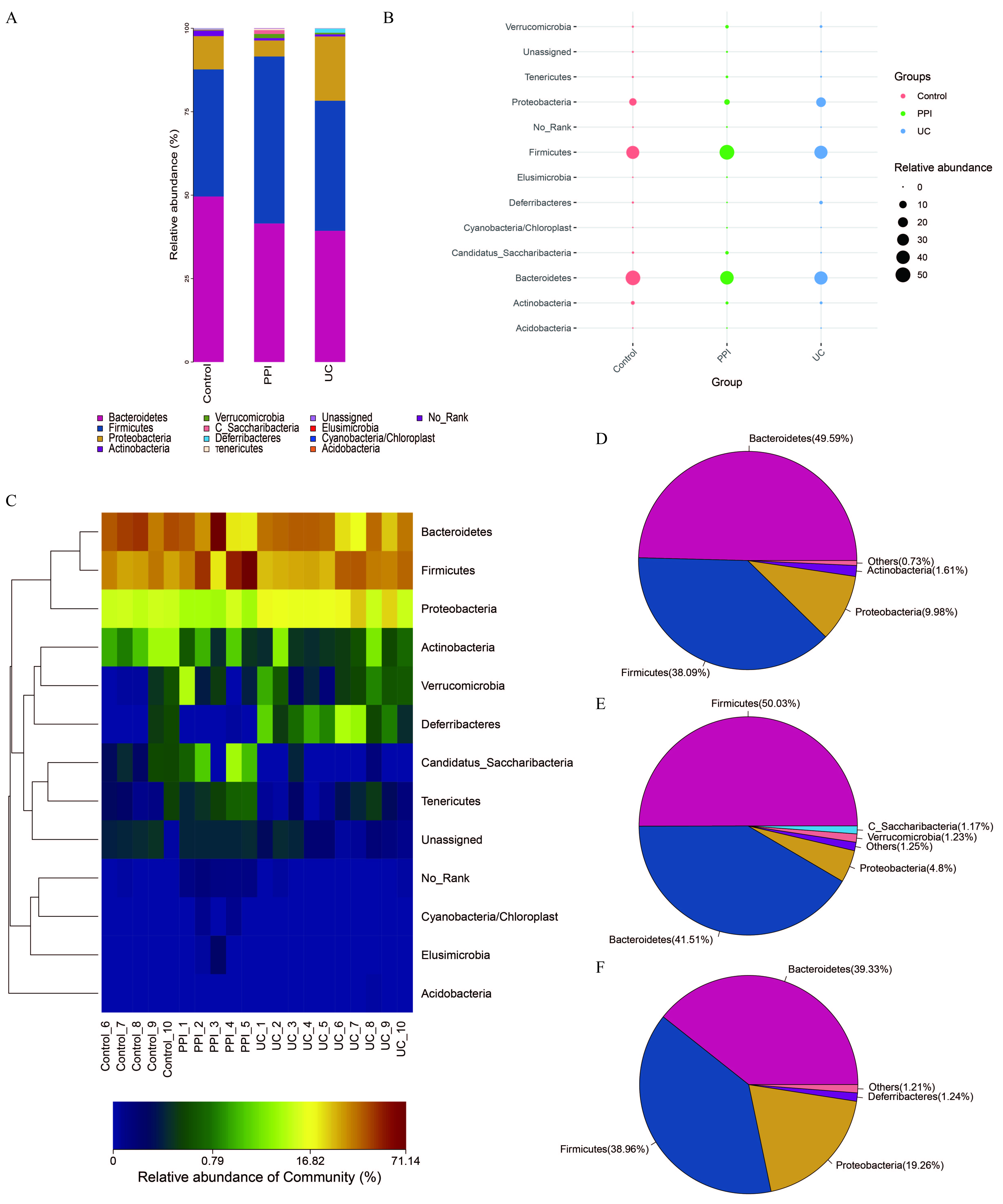

Supplement: SUPPLEMENTARY FIGURE S3 — Structure analysis of gut microbiota among control (n = 5), PPI (n = 5), and UC (n = 10) groups at the phylum level. (A) Barplot of species composition. (B) Bubble plot of abundance distribution. (C) Heatmap of sample clustering. (D) Proportions of main bacteria of the control group. (E) Proportions of main bacteria of the PPI group. (F) Proportions of main bacteria of the UC group. Barplot: Each bar represents a group; the vertical axis represents the relative abundance value. The average relative abundance of all species in each group adds up to 1, and each color corresponds to one species. Bubble: The horizontal axis represents the groups, the vertical axis represents the high-abundance species, and the size of the dots represents the relative abundance of the species in the sample. Heatmap: The horizontal axis represents the sample, the vertical axis represents the top 100 species with the highest abundance at the taxonomic level, and the color gradient from blue to red indicates the species abundance from small to large. Pie plot: Different colors represent different species, and the larger the fan area, the higher the abundance of that species. [file Image_3.jpeg]

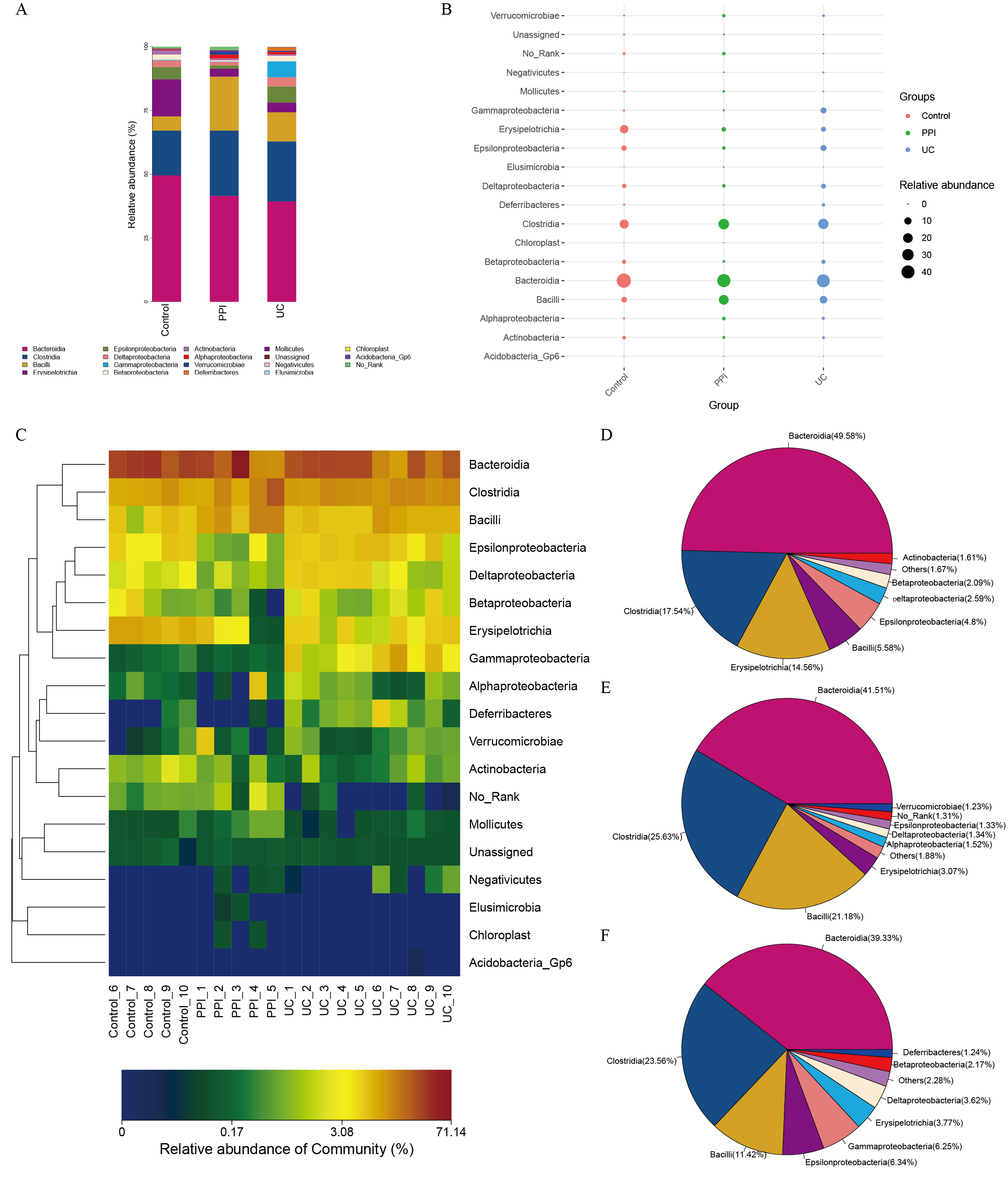

Supplement: SUPPLEMENTARY FIGURE S4 — Structure analysis of gut microbiota among control (n = 5), PPI (n = 5), and UC (n = 10) groups at the class level. (A) Barplot of species composition. (B) Bubble plot of abundance distribution. (C) Heatmap of sample clustering. (D) Proportions of main bacteria of the control group. (E) Proportions of main bacteria of the PPI group. (F) Proportions of main bacteria of the UC group. [file Image_4.jpeg]

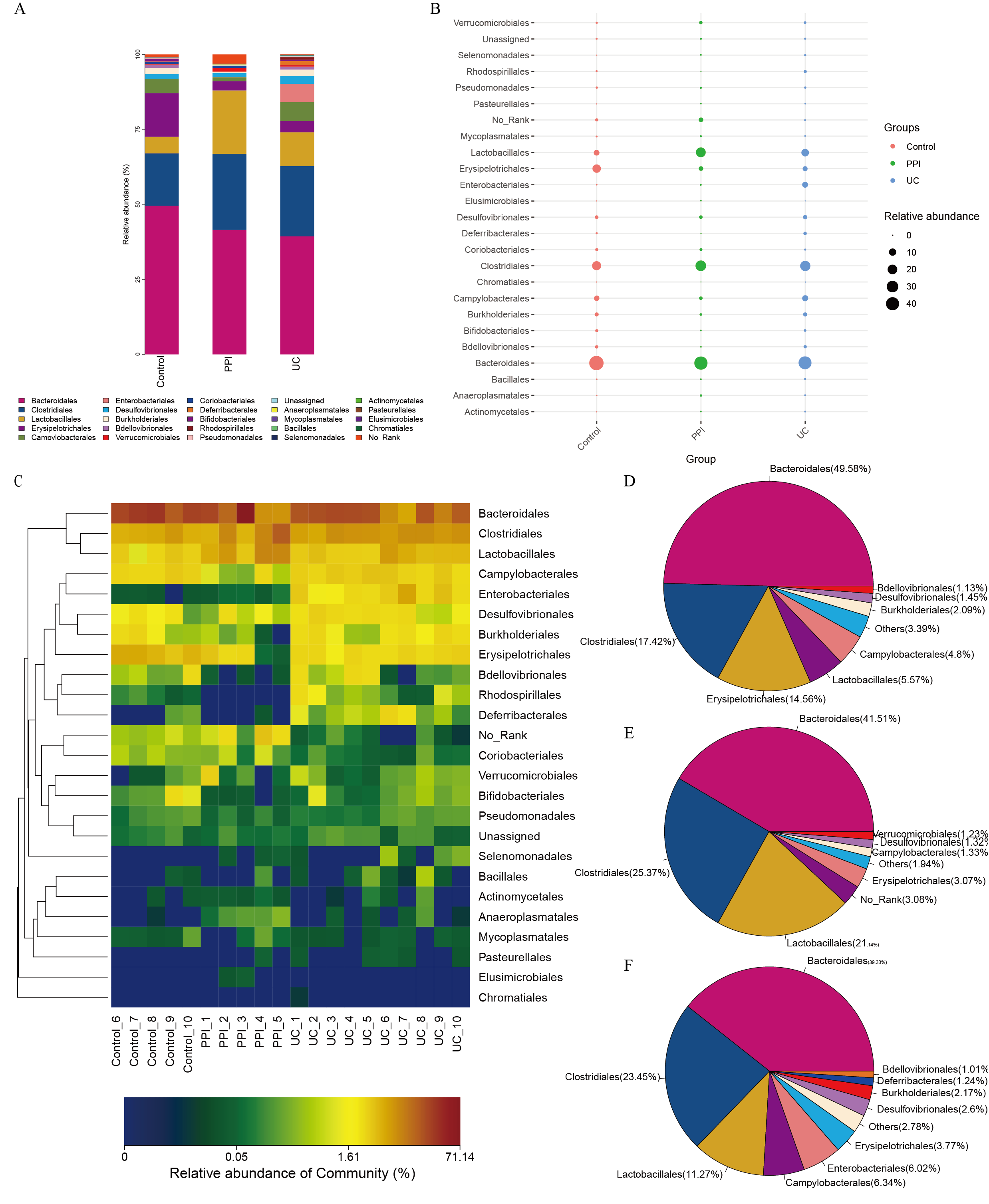

Supplement: SUPPLEMENTARY FIGURE S5 — Structure analysis of gut microbiota among control (n = 5), PPI (n = 5) and UC (n = 10) groups at the order level. (A) Barplot of species composition. (B) Bubble plot of abundance distribution. (C) Heatmap of sample clustering. (D) Proportions of main bacteria of the control group. (E) Proportions of main bacteria of the PPI group. (F) Proportions of main bacteria of the UC group. [file Image_5.jpeg]

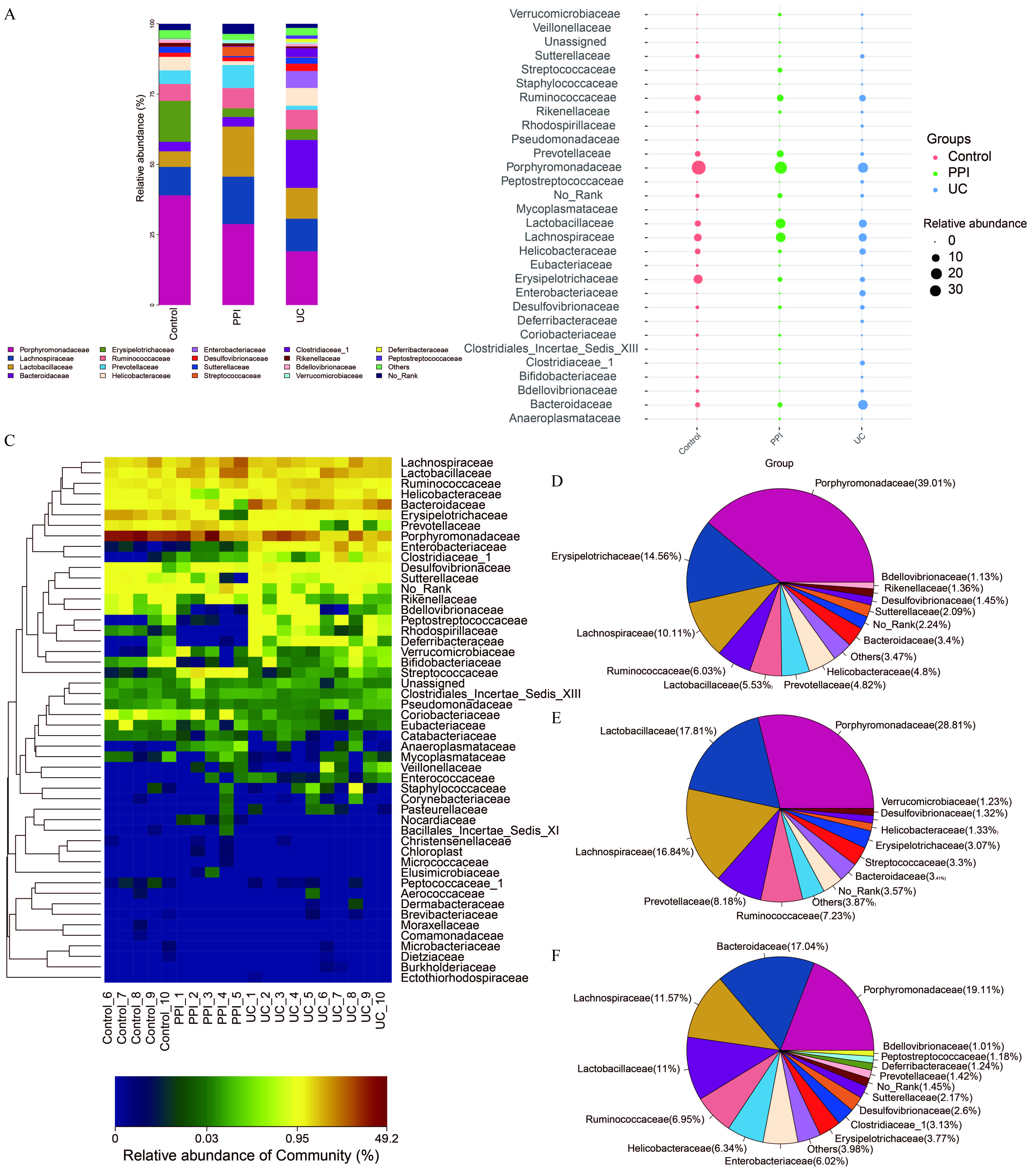

Supplement: SUPPLEMENTARY FIGURE S6 — Structure analysis of gut microbiota among control (n = 5), PPI (n = 5), and UC (n = 10) groups at the family level. (A) Barplot of species composition. (B) Bubble plot of abundance distribution. (C) Heatmap of sample clustering. (D) Proportions of main bacteria of the control group. (E) Proportions of main bacteria of the PPI group. (F) Proportions of main bacteria of the UC group. [file Image_6.jpeg]

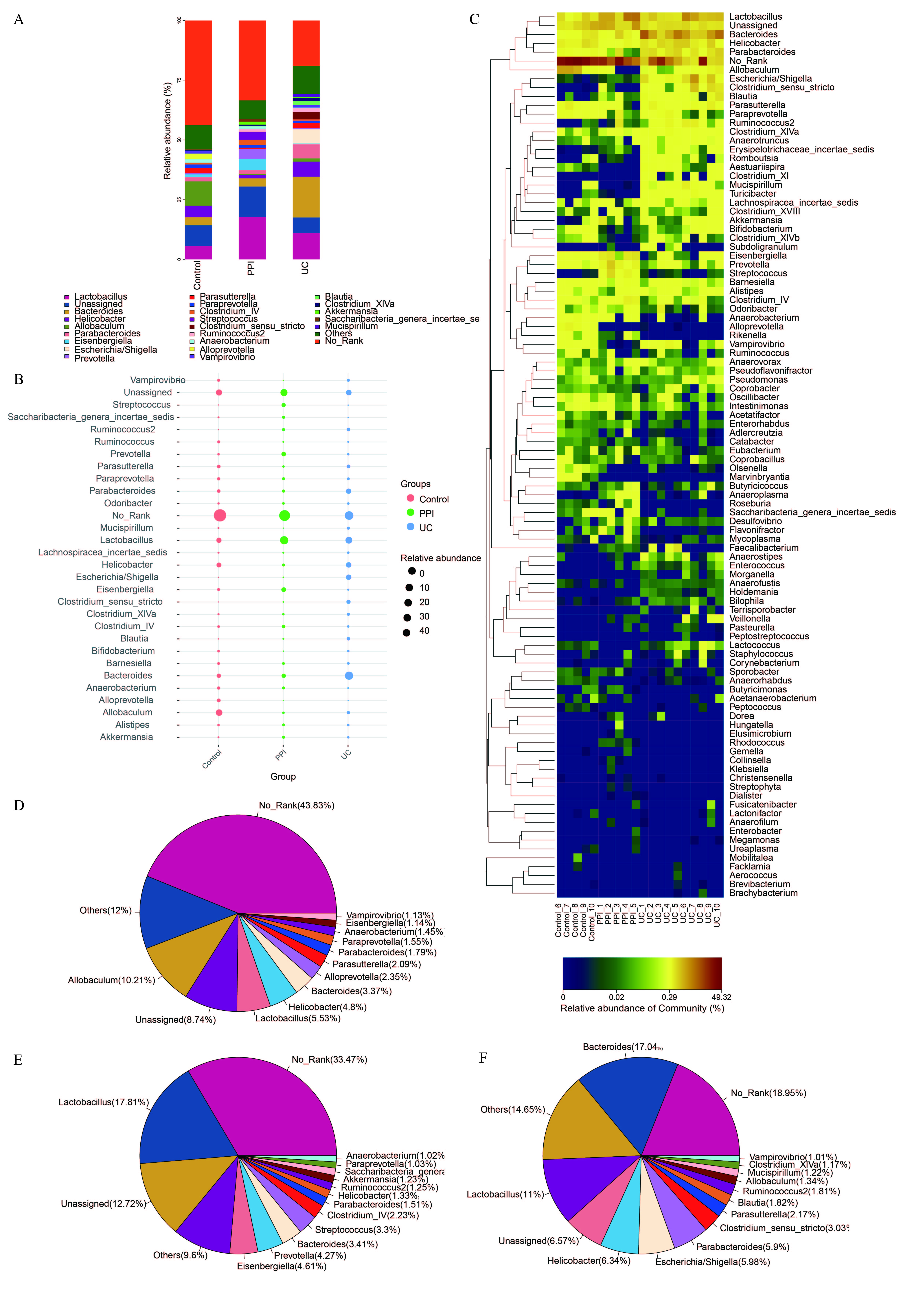

Supplement: SUPPLEMENTARY FIGURE S7 — Structure analysis of gut microbiota among control (n = 5), PPI (n = 5), and UC (n = 10) groups at the genus level. (A) Barplot of species composition. (B) Bubble plot of abundance distribution. (C) Heatmap of sample clustering. (D) Proportions of main bacteria of the control group. (E) Proportions of main bacteria of the PPI group. (F) Proportions of main bacteria of the UC group. [file Image_7.jpeg]

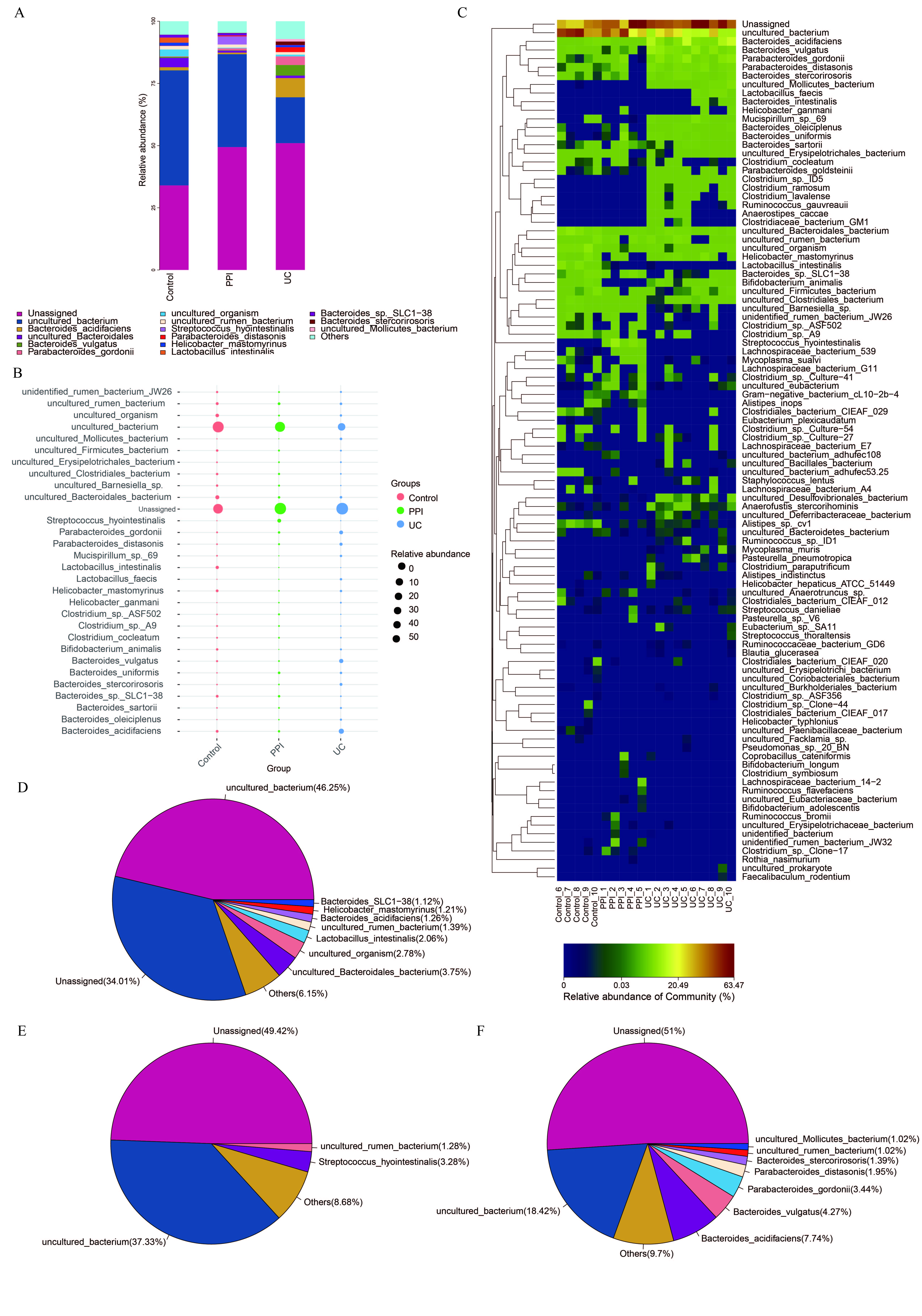

Supplement: SUPPLEMENTARY FIGURE S8 — Structure analysis of gut microbiota among control (n = 5), PPI (n = 5), and UC (n = 10) groups at the species level. (A) Barplot of species composition. (B) Bubble plot of abundance distribution. (C) Heatmap of sample clustering. (D) Proportions of main bacteria of the control group. (E) Proportions of main bacteria of the PPI group. (F) Proportions of main bacteria of the UC group. [file Image_8.jpeg]

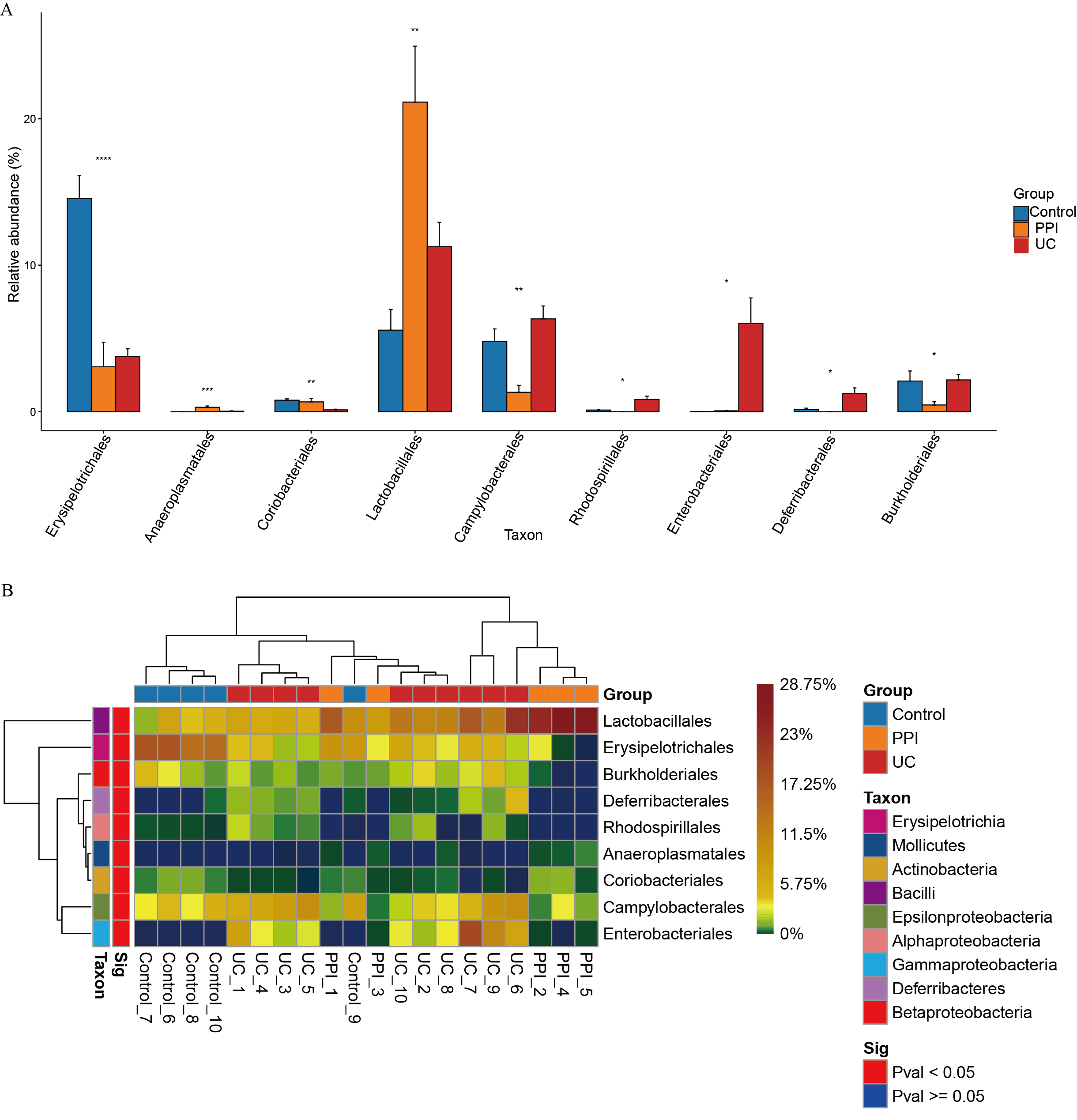

Supplement: SUPPLEMENTARY FIGURE S9 — Difference in composition of gut microbiota among control (n = 5), PPI (n = 5), and UC (n = 10) groups. (A) Barplot of differential microbial taxa at the order level. (B) Heatmap of differential microbial taxa at the order level. [file Image_9.jpeg]

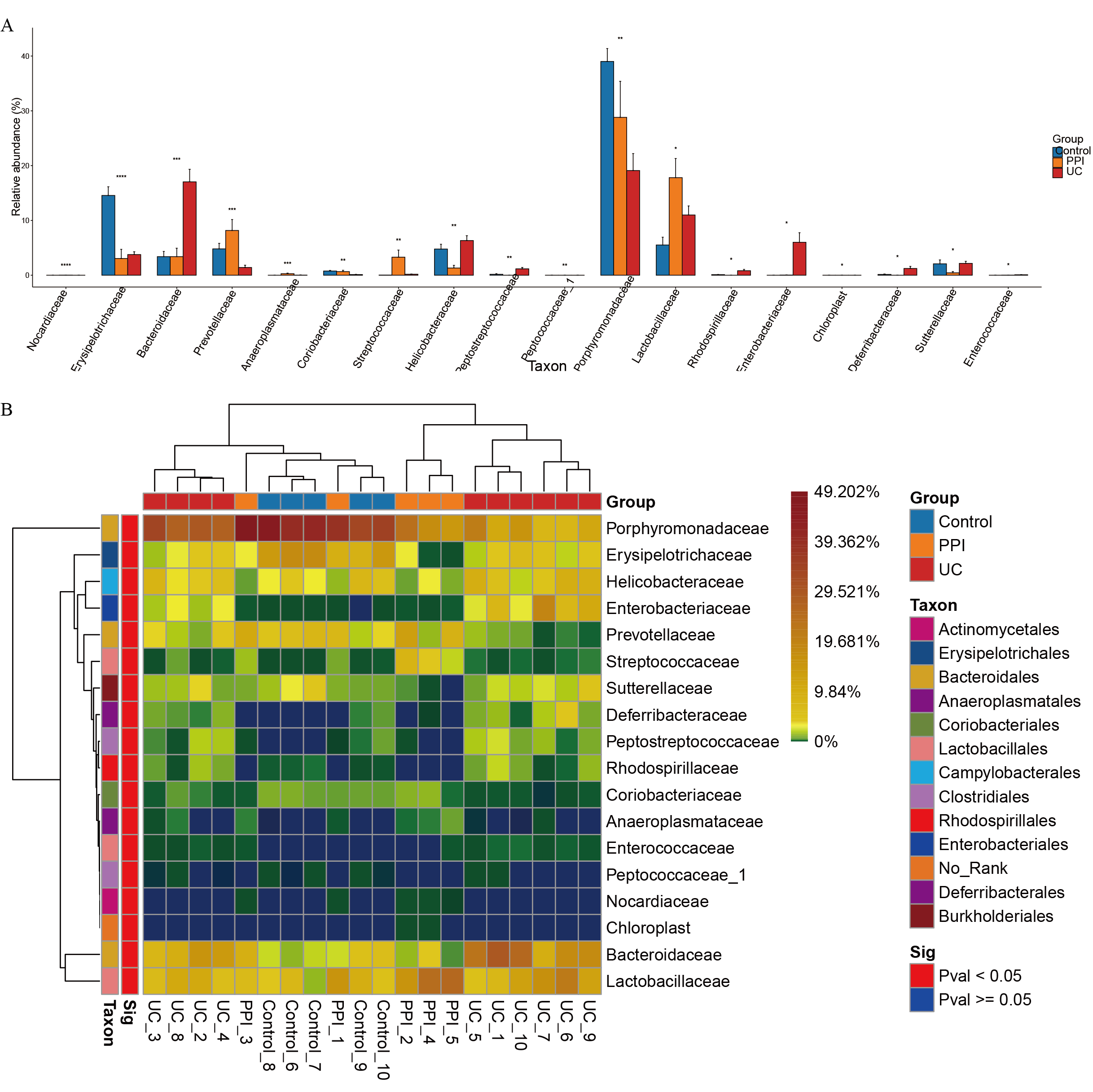

Supplement: SUPPLEMENTARY FIGURE S10 — Difference in composition of gut microbiota among control (n = 5), PPI (n = 5), and UC (n = 10) groups. (A) Barplot of differential microbial taxa at the family level. (B) Heatmap of differential microbial taxa at the family level. [file Image_10.jpeg]

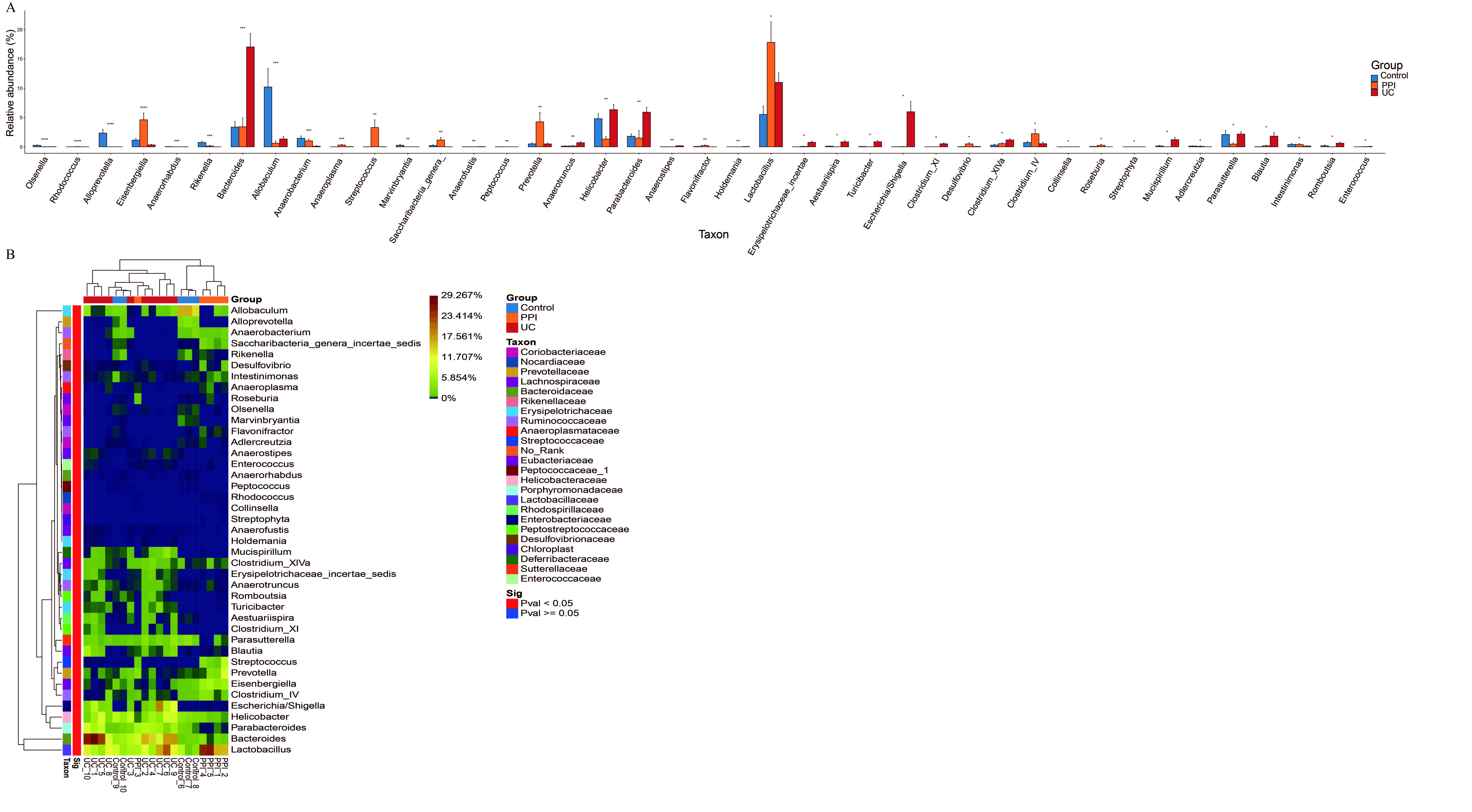

Supplement: SUPPLEMENTARY FIGURE S11 — Difference in composition of gut microbiota among control (n = 5), PPI (n = 5), and UC (n = 10) groups. (A) Barplot of differential microbial taxa at the genus level. (B) Heatmap of differential microbial taxa at the genus level. [file Image_11.jpeg]

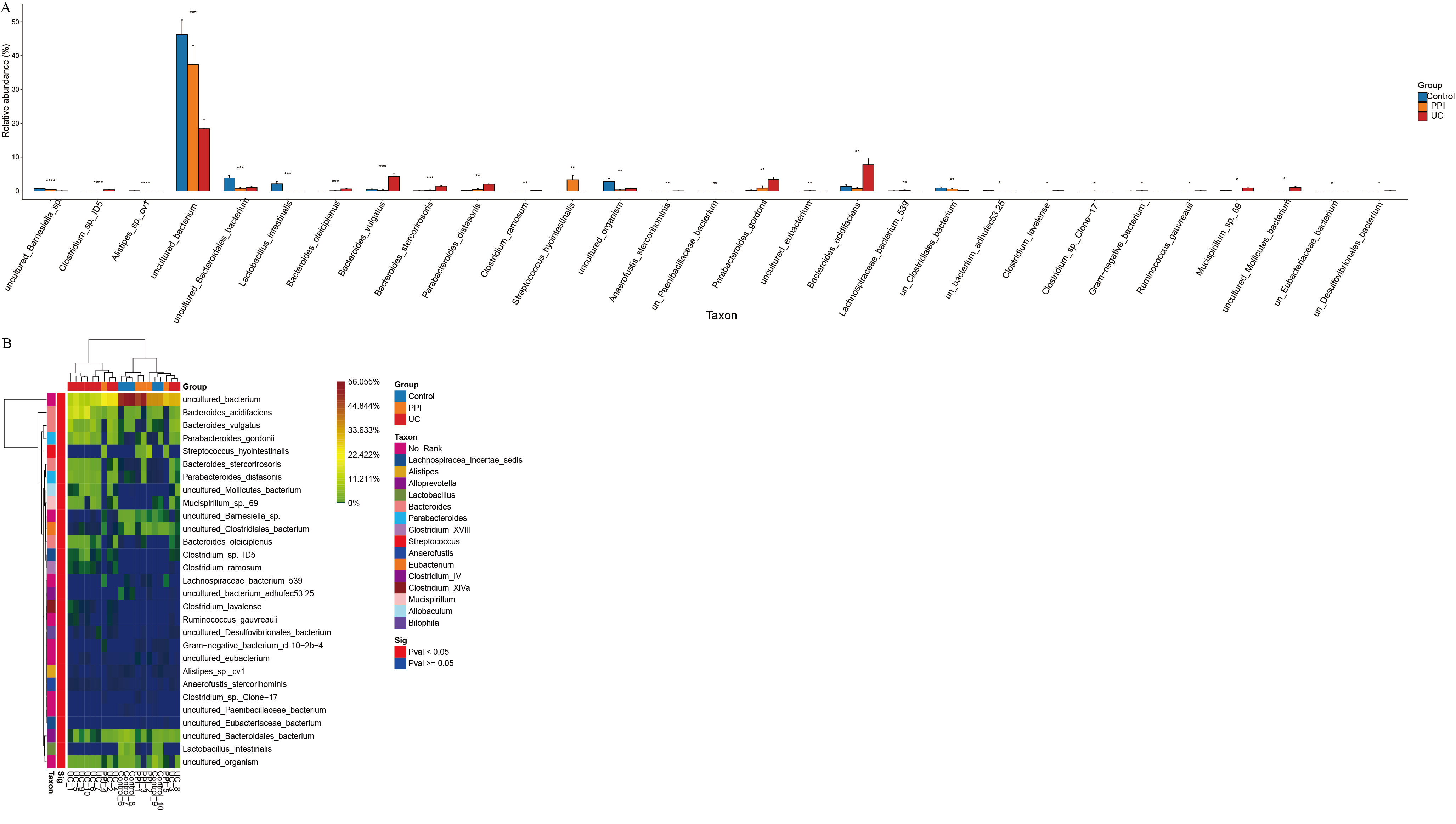

Supplement: SUPPLEMENTARY FIGURE S12 — Difference in composition of gut microbiota among control (n = 5), PPI (n = 5), and UC (n = 10) groups. (A) Barplot of differential microbial taxa at the species level. (B) Heatmap of differential microbial taxa at the species level. [file Image_12.jpeg]

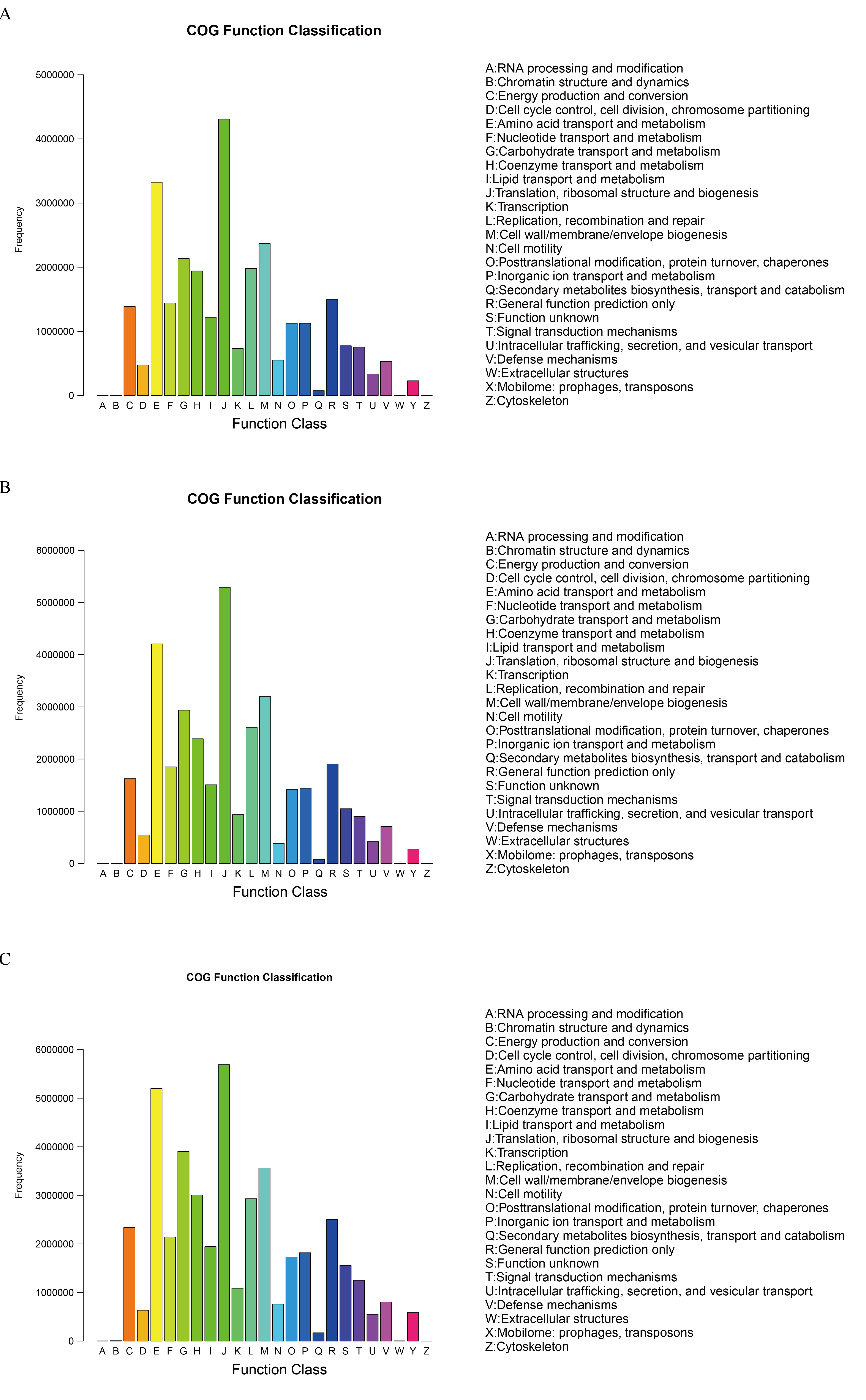

Supplement: SUPPLEMENTARY FIGURE S13 — Functional annotation and classification of samples from the control (n = 5), PPI (n = 5), and UC (n = 10) groups. (A) Control group. (B) PPI group. (C) UC group. The horizontal axis represents the Clusters of Orthologous Groups (COG) function, and the vertical axis represents the COG function abundance. [file Image_13.jpeg]

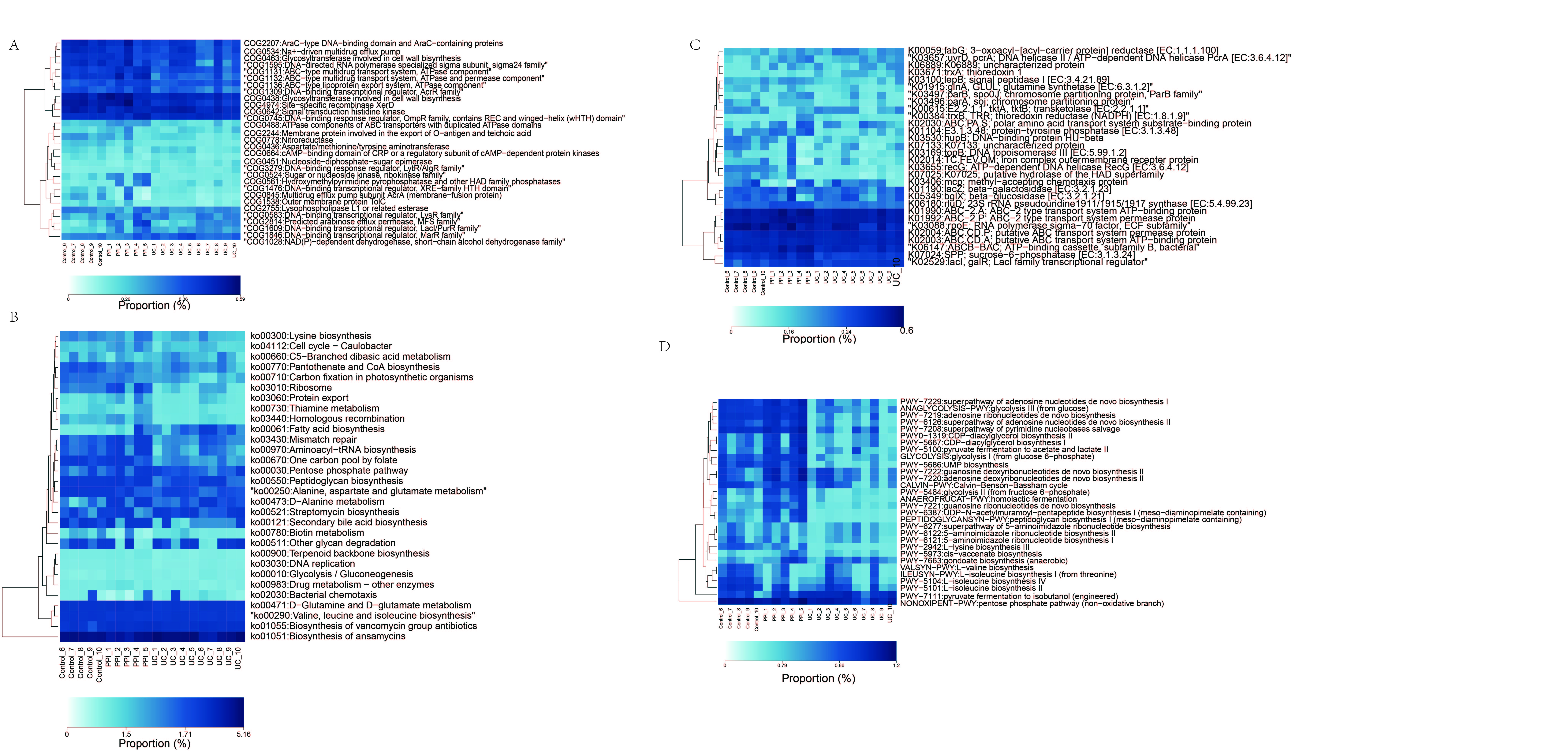

Supplement: SUPPLEMENTARY FIGURE S14 — Heatmap and clustering analyses of differences and similarities in the top 30 functions with the highest relative abundances among control (n = 5), PPI (n = 5), and UC (n = 10) groups. (A) Enrichment of functions annotated by Clusters of Orthologous Groups of proteins (COG). (B) Enrichment of functions annotated by clusters of Kyoto Encyclopedia of Genes and Genomes (KEGG). (C) Enrichment of functions annotated by clusters of KEGG Orthology (KO). (D) Enrichment of functions annotated by clusters of metabolic pathway database (MetaCyc). The horizontal axis represents the different sample, the vertical axis represents the top 30 functions with the highest abundance, and the gradient color from light to dark indicates the relative abundance of the functions from small to large. [file Image_14.jpeg]
