# Supplementary material for: Proton pump inhibitors and gut microbiota dysbiosis: insights into the pathogenesis of ulcerative colitis
Source: Front Microbiol. 2025 Oct 30;16:1657865. doi: 10.3389/fmicb.2025.1657865 (PMC12612687; doi:10.3389/fmicb.2025.1657865)
Supplement: Supplementary file 15 [file Table_1.DOCX]

**Table S1** The OTU annotation at different taxonomic levels for each sample

| Taxon | Superkingdom | Phylum | Class | Order | Family | Genus | Species |
| --- | --- | --- | --- | --- | --- | --- | --- |
| control_1 | 396 | 388 | 367 | 363 | 341 | 197 | 195 |
| control_2 | 427 | 416 | 404 | 398 | 381 | 195 | 207 |
| control_3 | 409 | 402 | 386 | 382 | 361 | 201 | 212 |
| control_4 | 546 | 540 | 516 | 513 | 495 | 261 | 282 |
| control_5 | 439 | 438 | 422 | 419 | 404 | 229 | 220 |
| PPI_1 | 289 | 284 | 276 | 275 | 261 | 154 | 150 |
| PPI_2 | 384 | 380 | 368 | 363 | 351 | 189 | 167 |
| PPI_3 | 222 | 215 | 215 | 213 | 203 | 132 | 110 |
| PPI_4 | 326 | 320 | 311 | 307 | 291 | 168 | 155 |
| PPI_5 | 380 | 374 | 366 | 364 | 355 | 184 | 170 |
| UC_1 | 322 | 311 | 308 | 306 | 295 | 205 | 134 |
| UC_2 | 360 | 348 | 346 | 342 | 333 | 201 | 160 |
| UC_3 | 481 | 473 | 461 | 458 | 433 | 255 | 221 |
| UC_4 | 350 | 346 | 346 | 344 | 327 | 207 | 165 |
| UC_5 | 329 | 325 | 321 | 318 | 304 | 202 | 150 |
| UC_6 | 219 | 218 | 217 | 216 | 213 | 157 | 97 |
| UC_7 | 233 | 231 | 231 | 230 | 226 | 170 | 101 |
| UC_8 | 423 | 418 | 413 | 409 | 392 | 224 | 202 |
| UC_9 | 272 | 268 | 268 | 264 | 252 | 169 | 115 |
| UC_10 | 285 | 280 | 280 | 278 | 270 | 193 | 138 |
