# Supplementary material for: Proton pump inhibitors and gut microbiota dysbiosis: insights into the pathogenesis of ulcerative colitis
Source: Front Microbiol. 2025 Oct 30;16:1657865. doi: 10.3389/fmicb.2025.1657865 (PMC12612687; doi:10.3389/fmicb.2025.1657865)
Supplement: Supplementary file 16 [file Table_2.docx]

**Table S2** ANOSIM analysis of the inter-group and intra-group differences among control, PPI and UC groups

| Group | Statistic R | Significance |
| --- | --- | --- |
| All | 0.9801 | 0.0001 |
| Control vs PPI | 0.8880 | 0.0093 |
| Control vs UC | 0.9993 | 0.0005 |
| PPI vs UC | 0.9920 | 0.0004 |
